# Increased rainfall-runoff drives flood hazard intensification in Central Himalayan river systems

**DOI:** 10.1038/s41598-025-26815-2

**Published:** 2025-12-08

**Authors:** Ivo Pink, Sim M. Reaney, Richard J. Hardy, C. Isabella Bovolo

**Affiliations:** 1 Department of Geography, Durham University; Institute of Hazard, Risk and Resilience, Durham University, Durham, UK; 2 Department of Geography, Durham University, Durham, UK

**Keywords:** Climate sciences, Environmental sciences, Hydrology, Natural hazards, Water resources

## Abstract

The Central Himalayan floodplain is one of the most flood-affected regions of the world, but we have limited knowledge of climate-change impacts on fluvial floods. Here, we provide the first large-ensemble, regional climate-change impact assessment of design floods for the Karnali River in Nepal and China, based on high-resolution modelling, considering climatic, hydrological and statistical uncertainties, needed for building local mitigation strategies. Our simulations project increases in the 1% annual exceedance probability flood magnitude of + 40% (P_10/90_: 33/48%) (medium-emissions) and + 79% (P_10/90_: 54/82%) (high-emissions) for 2060–2099, with rainfall-runoff contributing ≥ 90% of the additional flood water. We show that the uncertainty in the projections is linked to the relative differences between the magnitudes of the largest and more common events and argue that it is important to include both atmospheric only general circulation models and earth system Models in ensembles to capture the range of event and model uncertainty.

**Supplementary Information:**

The online version contains supplementary material available at 10.1038/s41598-025-26815-2.

## Introduction

The Central Himalayan floodplain is one of the most flood-affected regions of the world^1^ with the September 2024 floods causing damages of 1% of Nepal’s GDP with 236 deaths and 8,400 people displaced^2^ and by 2050, flood damages are projected to account for 2.2% of annual GPD^3^. Heavy rainfall and pluvial floods are projected to increase in this region, with changes occurring at faster rates than the global average^4,5^. Fluvial flood response to climate change is more uncertain, due to the complex interactions between environmental, climatic and hydrological conditions^6^. Understanding this response and its driving processes is critical for the development of flood risk management strategies targeted at the catchment-scale.

The current understanding of the climate-change impact on Central Himalayan river floods is largely based on global-scale projections of Global Circulation Model (GCM) runoff projections^7^, hydrodynamic routing of GCM runoff projections^8,9^, or hydrological modelling using GCM climate projections^10^. While global studies are valuable for understanding regional trends, their validity on the catchment-scale is limited for several reasons: (i) the global application leads to a loss of performance at the catchment-scale (e.g. the observed direction of change is not captured by GCM runoff predictions for 40% of the catchments^7^; (ii) the projections are averaged over a coarse modelling grid, which restricts change impact assessments to large catchments^7^; (iii) the small-scale variability of the hydro-climatic system of mountainous catchments is not captured by the coarse modelling grid of GCMs^5,11^. Regional-scale assessments provide an improved representation of this variability due to finer modelling grids, but the studies investigating the Central Himalayas have used older generations of climate projections^12,13^, and/or do not capture appropriate design floods used for flood risk management purposes (e.g. 1% Annual Exceedance Probability (AEP)). Furthermore, the studies that use Flood Frequency Analysis (FFA) to determine design flood magnitudes do not estimate the uncertainties introduced by the FFA, although this is also needed to correctly design flood mitigation strategies^14^.

**Fig. 1:**
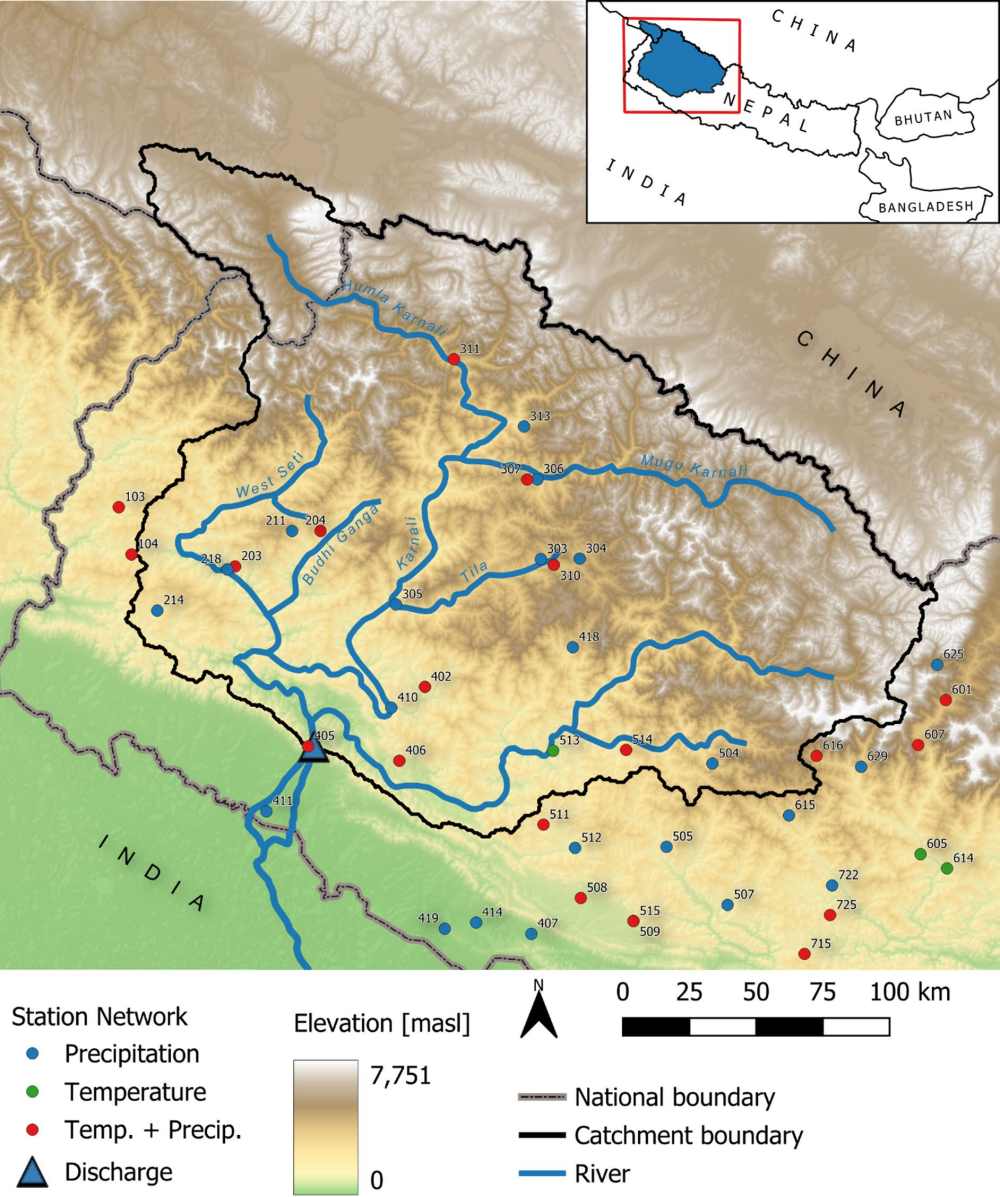
Map of the Karnali River catchment in Nepal and China. The topography is derived from the SRTM 90 m V4.1^67^. The station network shows the location of the hydrological and meteorological stations used in this study. The numbers on the station network relate to the gauge’s identification. These stations are maintained by the Department of Hydrology and Meteorology (DHM), Nepal. The map was created with QGIS Version 3.34.3 (https://qgis.org/) and uses the WGS84 UTM Zone 44° N coordinate system.

Here, we present the first, comprehensive regional climate change impact assessment of design flood hazards in the Central Himalayan Karnali River in Nepal and China (Fig. 1) derived from high-resolution, large ensemble modelling using the latest generation of climate projections, and capturing climatic, hydrological and statistical uncertainties. The applied framework couples the climate projections with a continuous, fully distributed (500 × 500 m) hydrological model and a FFA. We apply: probabilistic climate projections (12 bias-corrected and downscaled CMIP6 models^15^ for medium (SSP245) and high emission (SSP585) scenarios for the near-future (2020–2059) and far future (2060–2099) to estimate the climatic uncertainty; a Generalised Likelihood Uncertainty Estimation framework (GLUE)^16^ with 64 parameter sets to capture the hydrological uncertainty, and utilise the GLUE predictions to capture the measurement uncertainty; and a bootstrapping framework^17^ to capture the sampling uncertainty in the FFA. We apply the ‘Spatial Processes in Hydrology’ (SPHY) hydrological model^18^ which represents cryospheric and hydrological processes, including the simulation of glacier retreat^19^, which improves predictions for long-term climate change assessments. However, the current land cover is maintained throughout the modelling period as no high-resolution projections of potential future land cover exist for the Karnali catchment, and estimating the potential future land cover change would increase the uncertainty in the model predictions. Thus, the flood hazard projections do not account for potential land use changes.

Our simulations find, with high confidence, that flood magnitudes for the 1% AEP will increase by 23% and 26% in the near-future (2020–2059), and increase further in the far-future (2060–2099) by 40% and 79% for the medium and high emissions scenarios respectively. We also show that rainfall-runoff is the largest source of flood water (> 90%), but that snowmelt is an important source for individual medium-discharge flood events. We find that the FFA uncertainty is strongly affected by the timing and occurrence of rare precipitation events, which dominate flood magnitude response. We therefore argue that a large ensemble of climate models is needed to capture a large enough sample size of rare events to enable the analysis of flood frequencies under future climates. Both Atmosphere Only General Circulation Models (AOGCMs) and Earth System Models (ESMs) should be included in the ensemble to capture climate model uncertainty, as AOGCMs tend to project a wider range of changes with respect to the baseline, with lower minimum bounds, whilst ESMs project a narrower range of and higher median changes. These results build on existing studies to provide an advance in understanding of fluvial flood uncertainty and drivers, and propose a modelling framework for predicting AEP flood magnitudes under climate change, necessary for flood risk mitigation.

### Projected changes in flood magnitudes

**Fig. 2:**
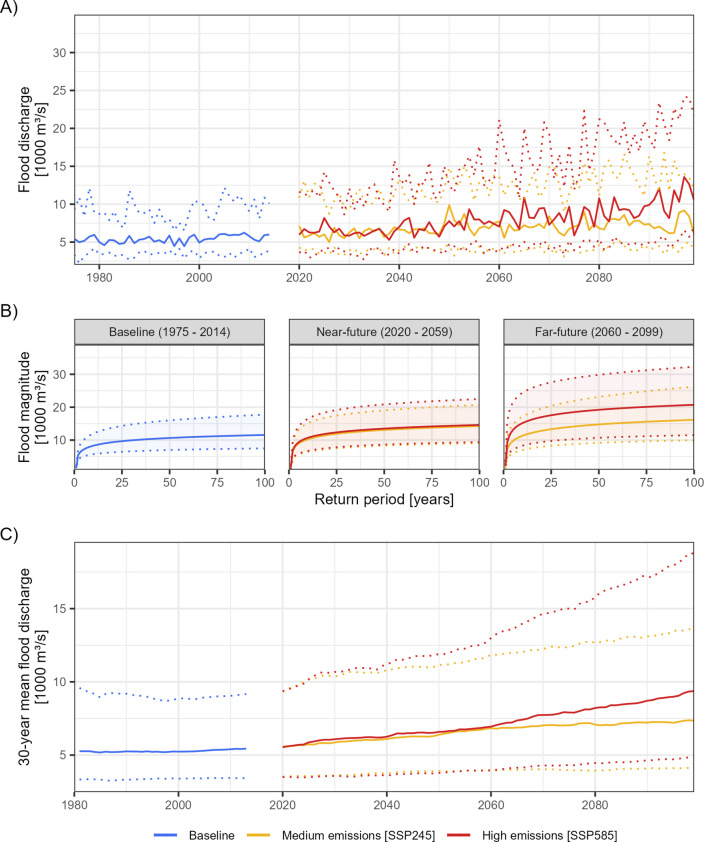
The flood hazard simulations of the flood discharge classified as AMAX flows (**A**), the flood magnitudes predicted by the FFA (**B**), and the 30-year mean flood discharge (**C**), for the scenario and two projected climate scenarios predicted for probabilistic climate projections of 12 CMIP6 models. The solid lines indicate the median, and the dotted lines indicate the 10 and 90th percentiles of the ensemble.

Flood discharge projections obtained from hydrological modelling (Fig. 2a) are used in the FFA to estimate flood magnitudes for probabilistic climate projections. Our results show that in the near-future (2020–2059), the P_50_ flood magnitudes of events with a 1% AEP are projected to increase from the baseline (1975–2014) scenario by + 23% (P_10_ + 21%, P_90_ + 17%) for the medium-emission scenario, SSP245, and + 26% (P_10_ + 25%, P_90_ + 27%) for the high-emission scenario, SSP585 (Fig. 2b), with 10 (SSP245) and 11 (SSP585) of the 12 climate models projecting increases. However, the ensemble-median difference between both scenarios is 3%, with higher increases for SSP245 than for SSP585 for 6 of the 12 climate models.

In the far-future (2060–2099), the P_50_ 1% AEP flood magnitude increases from the baseline by 40% (P_10_ + 33%, P_90_ + 48%) for SSP245 and 79% (P_10_ + 54%, P_90_ + 82%) for SSP585 (Fig. 2b) with 12 (SSP245) and 11 (SSP585) of the 12 climate models projecting increases. Similarly, the frequency of a given flood magnitude increases, with the P_50_ 1% AEP event projected to occur once every 11 years for SSP245 and every 3 years for SSP585, with 12 (SSP245) and 11 (SSP585) of the 12 climate models projecting increases. An intensification of magnitude occurs with higher emissions, with 11 of the 12 climate models projecting higher increases for SSP585 than for SSP245.

The timing when both emission scenarios diverge varies between the percentiles of the ensemble. The P_50_ 30-year-mean flood discharge increases at similar rates for SSP245 (+ 20%) and SSP585 (+ 21%) between the years 2020 and 2059 (Fig. 2c). In the year 2060, the P_50_ 30-year-mean flood discharge of both scenarios starts diverging, and it further increases by 7% (SS245) and 35% (SSP585) between 2060 and 2099. The timing when higher flood discharges start to be predicted for SSP585 varies with the percentile, the P_90_ starts diverging between 2035 and 2040, the P_50_ around the year 2060, and the P_10_ between 2065 and 2070.

**Fig. 3:**
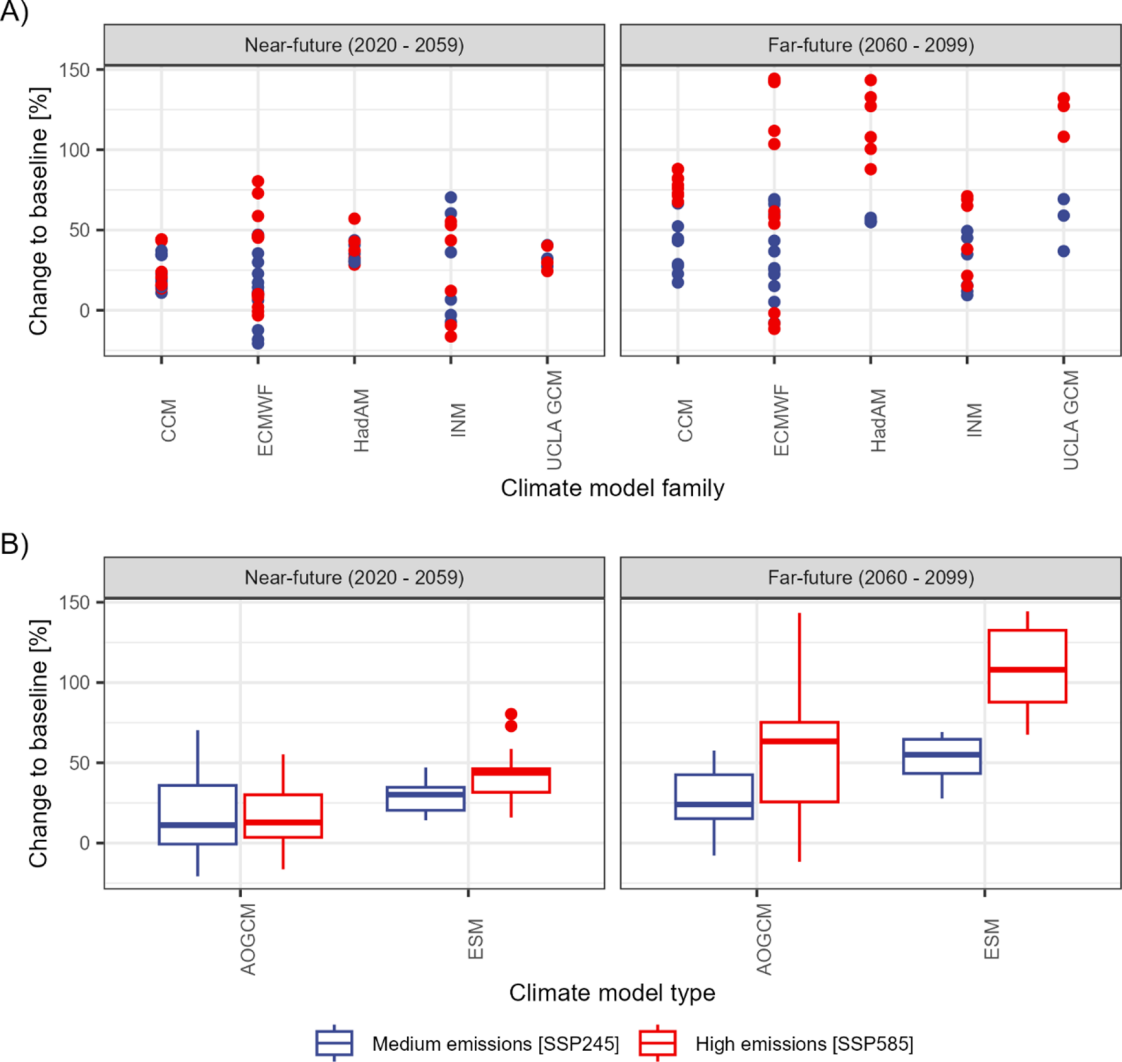
The projected changes of the median 1%-AEP flood magnitudes relative to the Baseline for the 12 CMIP6 members grouped by their climate model family (**A**); and grouped by the type of climate model into 6 atmospheric only general circulation models (AOGCMs) and 6 earth system models (ESMs) (**B**). The classification of model family and model type is based on Kuma et al.^20^.

We do not identify systematic patterns in the projected floods for related climate models^20^, despite models in the same family usually having similar climate properties; models of the same family can project both small and large changes in 1% AEP flood magnitudes (Fig. 3). Some models (e.g. HadAM, CCM) project smaller spreads, whilst others project larger spreads (e.g. ECMWF). Nevertheless, we find that ESMs generally project larger increases and provide a smaller range of change than AOGCMs, possibly related to their inclusion of the carbon cycle, vegetation, atmospheric chemistry and other processes, although other comparisons accounting for differences between AOGCMs and Earth System Models (ESMs) are currently lacking^20^, and further testing is necessary to confirm this result.

### The sources of floodwater

**Fig. 4:**
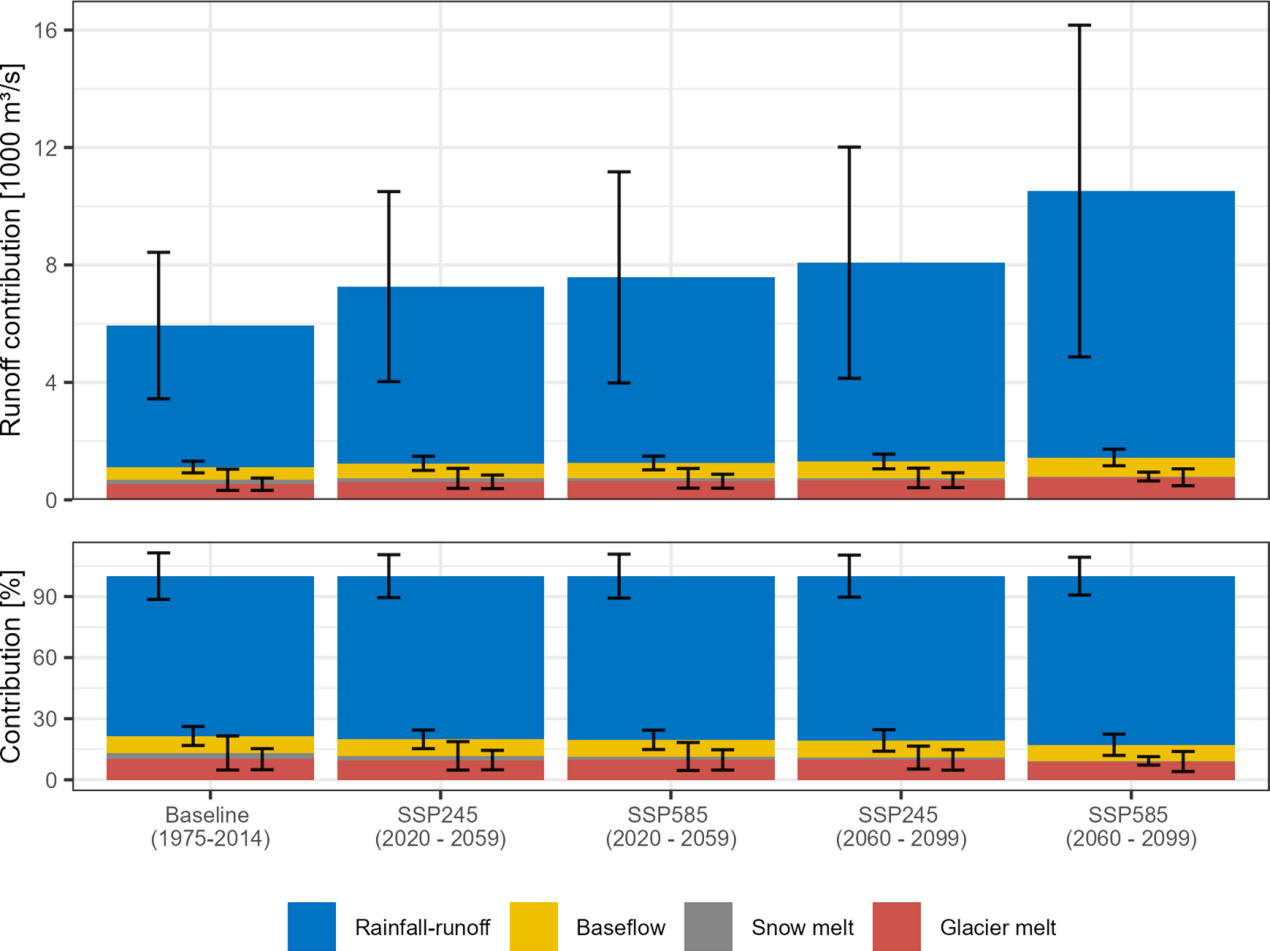
The mean flood discharge composition. The error bars indicate the standard deviation, which is caused by differences in the event characteristics, climate projections, and the hydrological model parameterisation.

**Fig. 5:**
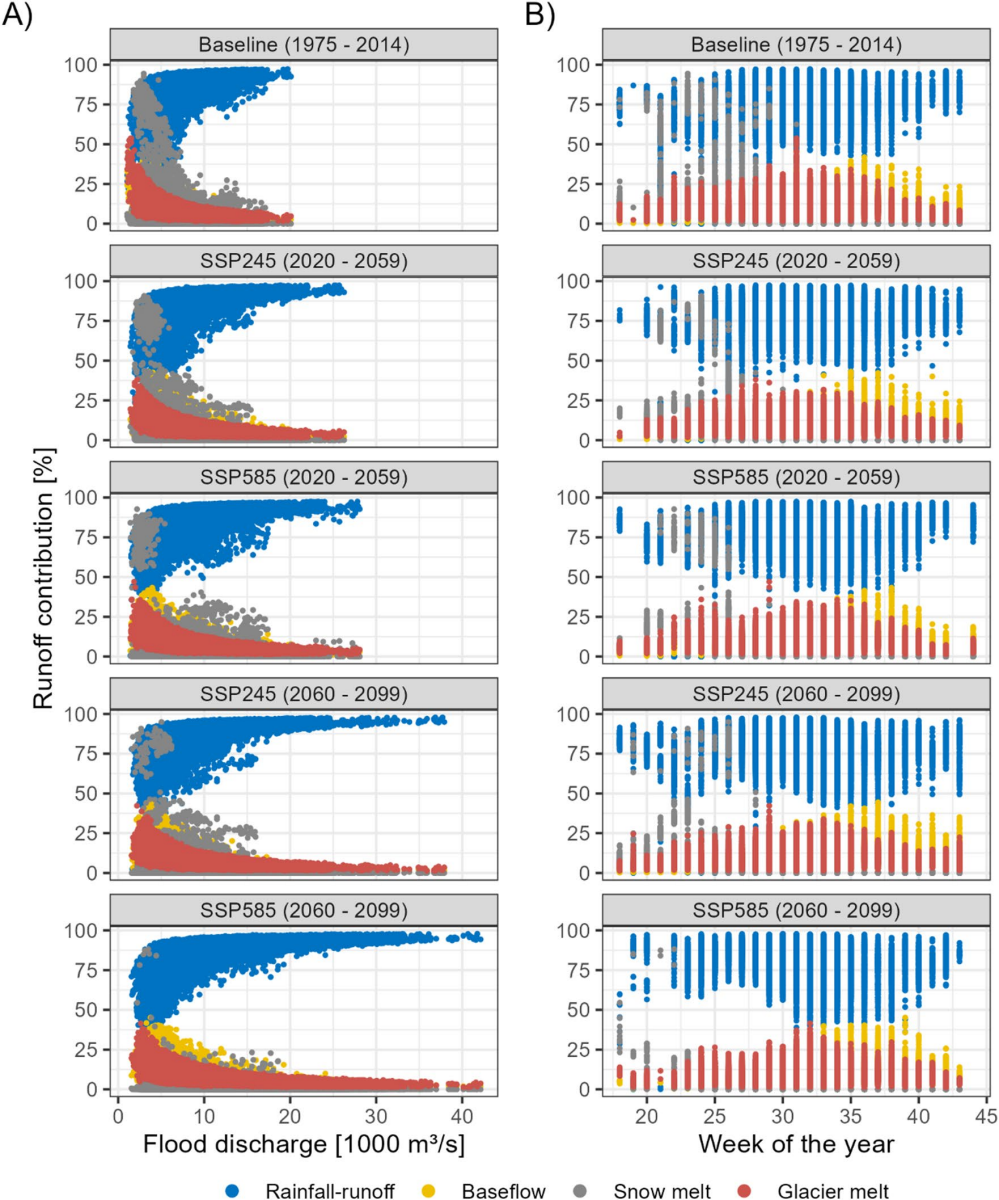
Composition of each simulated flood event (*n* = 12 Climate models x 64 hydrological parameter sets x 40 years). Column A shows the relationship between the runoff contribution and the magnitude of the flood discharge. Column B shows the relationship between the runoff contribution and the timing of the flood event.

We analyse the floodwater sources simulated by the hydrological model, divided between direct rainfall-runoff, glacier melt, groundwater-driven baseflow and snowmelt, to understand the drivers of the projected flood intensification. In the baseline period, rainfall-runoff is the most important source, with a mean floodwater contribution of 78 ± 11% (± standard deviation), followed by glacier melt (10 ± 5%), baseflow (8 ± 5%) and snowmelt (3 ± 8%) (Fig. 4). The importance of rainfall-runoff increases with the flood discharge, with rainfall-runoff contributing ≥ 90% to events with discharges ≥ 17,500 m^3^/s (high flows) (Fig. 5a). Consequently, the importance of the other sources decreases with increasing discharge. However, other sources are important for events with lower (< 10,000 m^3^/s) and medium (10,000–17,500 m^3^/s) discharges with contributions ≥ 25%, and 10–25% respectively (Fig. 5a). High rainfall-runoff contributions are predicted without clear seasonal patterns, but event timing is important: higher snowmelt contributions are predicted in the pre- and early-monsoon seasons (typically up to the 27th week of the calendar year); glacier meltwater contributions increase towards the mid-monsoon season (weeks 27–35); and higher baseflow contributions are predicted in the late-monsoon and post-monsoon seasons (week 35 onwards). These results provide a new contribution to the literature.

### The drivers of change

In the near-future and far-future, the projected flood intensification is driven by increasing rainfall-runoff for both scenarios. The mean flood discharge increases by 1,380–4,581 m^3^/s compared to the baseline, whereas the mean rainfall-runoff increases between 1,202 and 4,255 m^3^/s, and thus accounts for 91–93% of the projected increase of the mean flood discharge (Fig. 4). Furthermore, increasing rainfall-runoff also causes the intensification of high flood discharges, which remain composed of ≥ 90% rainfall-runoff (Fig. 5a). The baseflow and glacier melt contributions to the mean flood discharge increase by 77–215 m^3^/s and 82–215 m^3^/s, respectively (Fig. 4). Their relative importance decreases due to the higher rainfall-runoff increase, and we project no changes in their seasonality. The flood discharge intensification is dominated by increasing rainfall-runoff in both emissions scenarios, and thus, differences in future emissions affect the rate of change rather than their driving processes.

Temperature-related changes in melt processes lead to a decrease in snowmelt runoff for most flood events. The mean snowmelt contribution decreases from 3 ± 8% in the baseline to 2 ± 7% in the near-future (SSP245 and SSP585) and 1 ± 6% (SSP245) and 0 ± 2% (SSP585) in the far-future, which is caused by an earlier onset of the melting season (Fig. 5b). However, the occurrence probability of medium-discharge flood events with 10–40% snowmelt contributions is projected to increase from 0.30% in the baseline to 0.65% in the near-future, after which it decreases to 0.17% for SSP585. For SSP245, an increase to 0.45% is delayed to the far-future. The changes in the frequency of these events indicate that rising temperatures cause an initial increase in melt contributions for individual pre-monsoon and early monsoon season flood events. The warming also shifts the melting season earlier, which leads to lower flood discharge contributions as the temperature continues to increase, as indicated by the lower frequency in the far-future of SSP585 (Fig. 5a). While the occurrence probability is below 1% at the mountain outlet, the projected changes may indicate a shift in the flood seasonality, frequency and magnitude in smaller upstream subbasins.

### Performance of the hydrological model

**Fig. 6:**
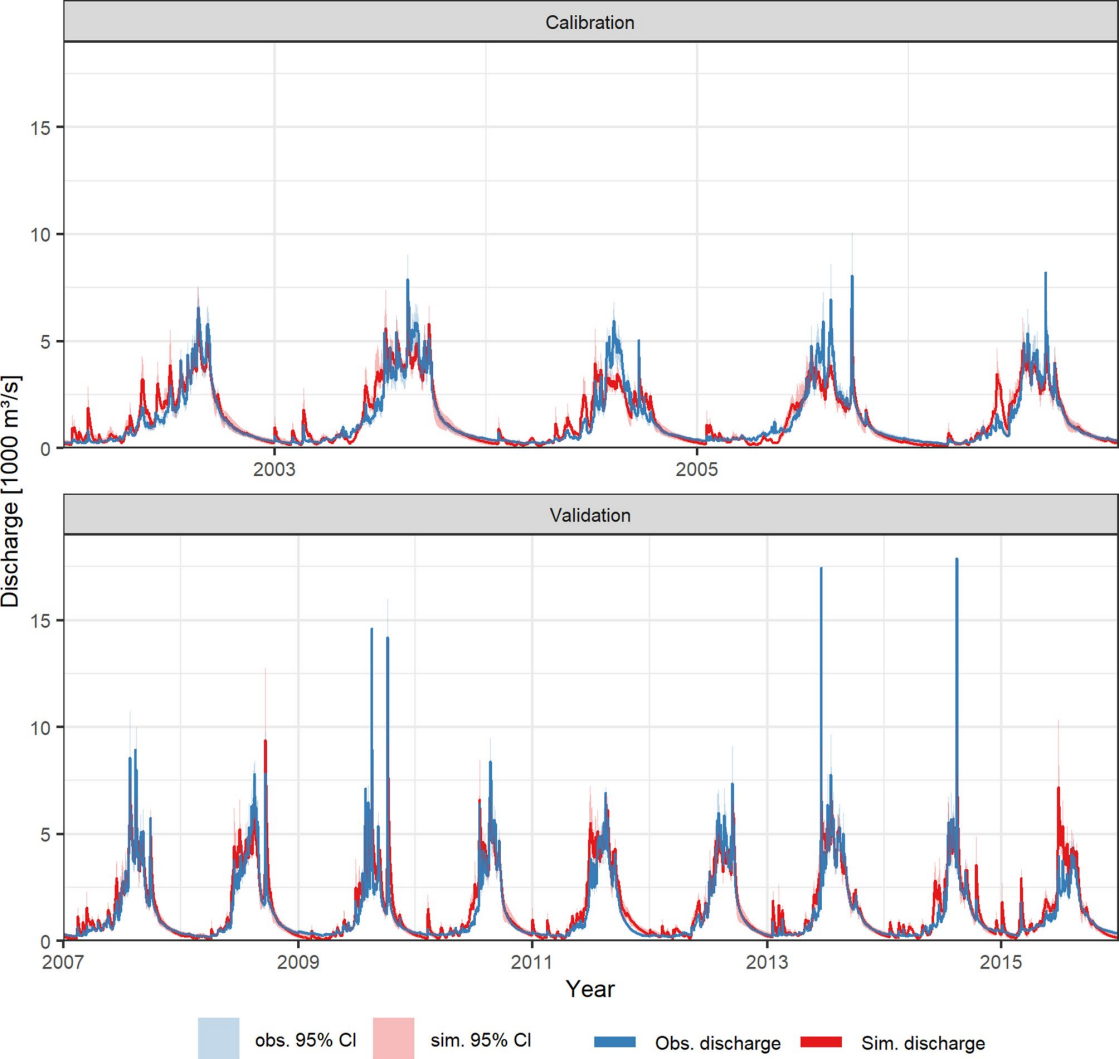
Hydrographs of the Karnali River for the calibration and validation periods. The discharge observations are obtained by the Department of Hydrology and Meteorology Nepal (DHM). The solid lines represent the ensemble-median (simulations) and the observations. The intervals represent the 2.5th to 97.5th interval of the ensemble predictions, and the discharge uncertainty of the observations. The median Nash-Sutcliffe efficiencies are 0.85 and 0.82 for the calibration and validation periods, respectively.

The hydrological ensemble is a behavioural representation of the hydrological system of the Karnali River catchment, indicated by the hydrograph inspection, the model efficiencies and the streamflow composition. The ensemble replicates the flow seasonality with high flows during the monsoon and low flows during the winter and times the transitions in the Pre-monsoon and Post-monsoon seasons well (Fig. 6). The seasonality of the runoff composition (Supplementary Figure S4) with snowmelt contributions in the Pre- and early Monsoon season (Mar – Jun), rainfall-runoff and glacier melt during the Monsoon season (Jun – Sep), and baseflow in the Post-monsoon season (Oct – Nov) reflects our understanding of Central Himalayan catchment hydrology^11,21–23^. The good model performance is, furthermore, illustrated by the high ensemble median efficiencies of: (i) the Nash-Sutcliffe-Efficiency (NSE) of 0.85 (range: 0.75–0.87) for the calibration period and 0.82 (range: 0.70–0.85) for the validation period, and (ii) the coefficient of determination (R^2^) of 0.85 (range: 0.77–0.87) and 0.84 (0.80–0.86) for the calibration and validation periods, respectively. These efficiencies are at the higher level of reported efficiencies in Central Himalayan catchments, ranging between 0.56 and 0.88 (NSE) and 0.63–0.89 (R^2^)^23–27^.

**Fig. 7:**
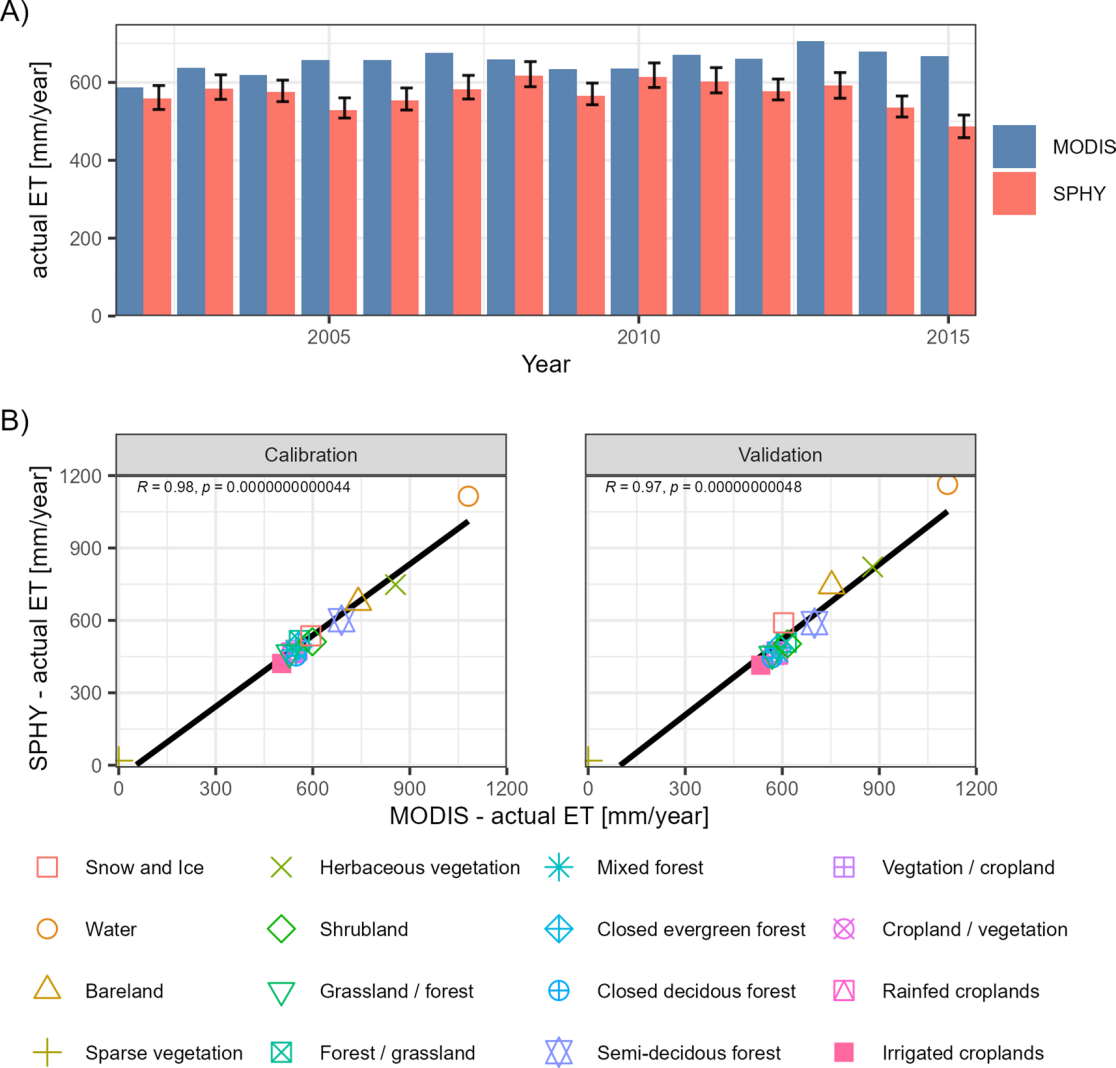
Comparison of the annual actual evapotranspiration (ET_a_)simulations with MODIS satellite estimates. (**A**) shows the catchment-mean ET_a_ rates, whereas the bars show the ensemble-median rates and the error bars the standard deviation. (**B**) compares the land-cover-specific mean ET_a_ of the ensemble-median and MODIS.

The evapotranspiration module was calibrated and validated against satellite-based annual actual evapotranspiration (ET_a_) estimates (Fig. 7a). The ensemble-median PBIAS is -11% (-20% to + 10%) in the calibration period and − 13% (-22% to + 7%) in the validation period, indicating a moderate underestimation of the catchment-mean ET_a_ rates. However, the performance of each ensemble member is satisfactory or better^28^, and the correlation between observed and simulated land-cover-specific ET_a_ rates is very high (R^2^ > 0.95), indicating a very good representation of the spatial ET_a_ variability (Fig. 7b). However, the model’s ability to reproduce ET_a_ rates varies between the years, and the percentage difference is within − 3% to -15%, but reaches up to -22% to -32% in the years 2005, 2014 and 2015. However, given the large uncertainty in the climate input datasets, these biases are likely caused by precipitation input deficits, as precipitation products are known to underestimate the high-mountain precipitation^29,30^. If Et_a_ observations are not considered in the model calibration, precipitation input deficits may be compensated by an ET_a_ underestimation^31,32^. Thus, we argue that it is important to consider ET_a_ observations during the calibration of hydrological models in Himalayan catchments.

### The uncertainty in the modelling ensemble

We analyse the uncertainty composition of the ensemble predictions using the 3-way analysis of variance (ANOVA) framework^33^. However, instead of decomposing the uncertainty contribution of climate models, climate scenarios and the internal variability, we assess the contribution of the ensembles of climate models, hydrological parametersets, and flood frequency curves. It is worth noting that the FFA ensemble estimates the uncertainty associated with the internal variability from a bootstrapping approach^17^.

**Fig. 8:**
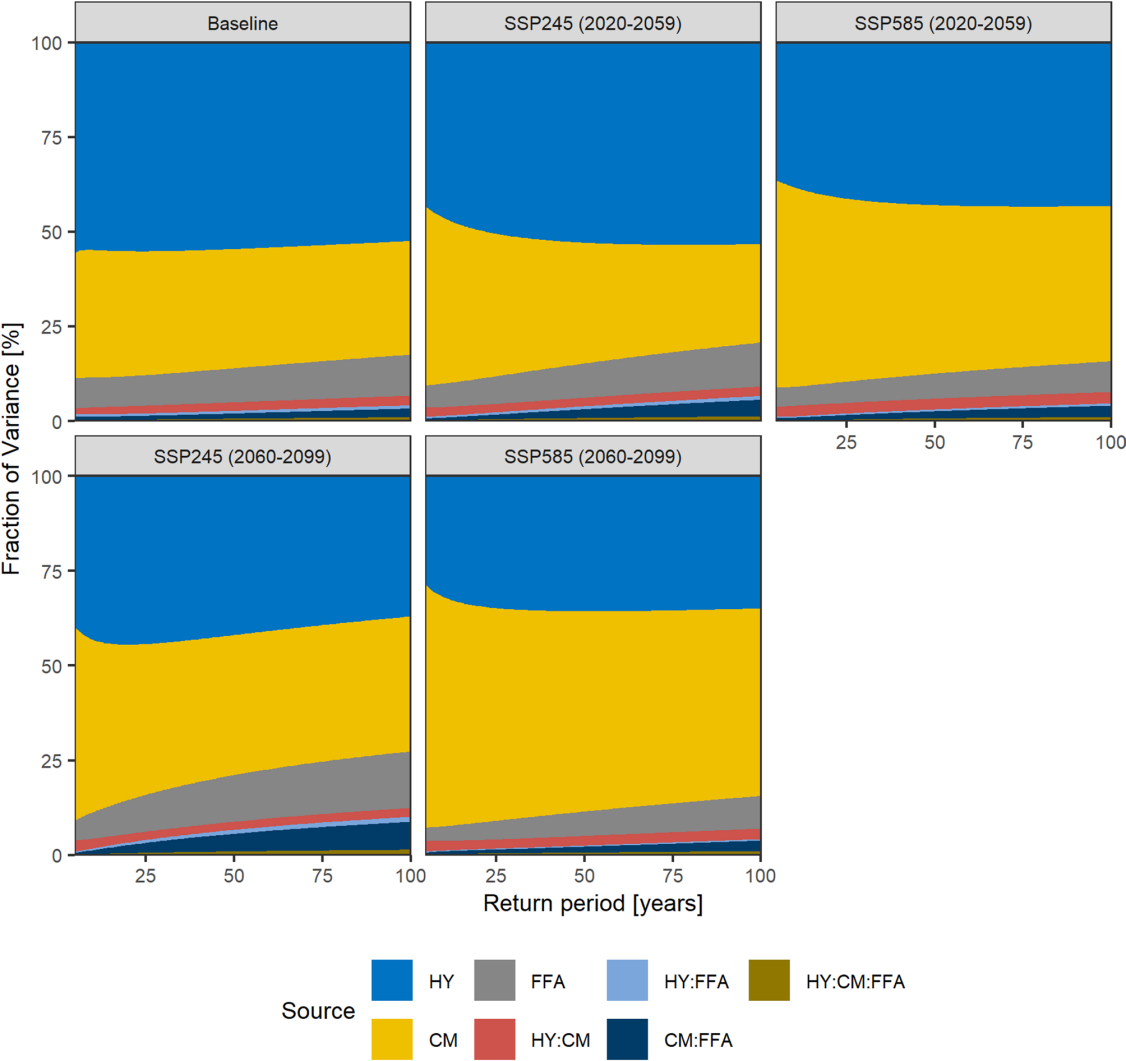
The uncertainty composition of the modelling ensemble estimated from 3-Way ANOVA. The main uncertainty components are the hydrological parametersets (HY), the climate model ensemble (CM) and the flood frequency analysis ensemble (FFA). The p-values of all main components and HY: CM interaction effects are below 0.05. The p-values of the FFA component interactions are > 0.1 with exception of the CM: FFA interactions for SSP245 (2060–2099) (< 0.1) (Supplementary Table S5).

The hydrological ensemble is the largest uncertainty source in the baseline, contributing 52% to the overall variance of the 1% AEP event (Fig. 8). For the projected scenarios, the hydrological uncertainty varies between the climate scenarios, contributing 53% (SSP245) and 43% (SSP585) to the 1% AEP in the near-future, and 37% (SSP245) and 35% (SSP585) in the far-future. However, the mean standard deviation (σ) of the hydrological ensemble averaged over the climate ensemble remains with 20–22% stable for all climate scenarios and return periods (Supplementary Figure S 5). This indicates that the hydrological parametersets introduce a large, but relatively constant uncertainty, and that the decreasing relative variance contribution reflects an increase in the overall variance.

The variance introduced by the climate models increases with time and emissions, but this trend is superimposed by an increasing FFA variance for higher frequencies. The climate model ensemble contributes 30% to the overall variance of the 1% AEP in the baseline. For SSP245, this fraction decreases to 26% in the near-future, after which it increases to 36% in the far-future. For SSP585, the contribution is with 41% (near-future) and 49% (far-future) notably higher than for SSP245, which indicates that the flood response becomes increasingly uncertain with time and emissions. The initial decrease in the climate ensemble variance for the near-future of SSP245 is caused by superimposing effects of the FFA variance, which is indicated by consistent climate variance increases with time and emissions for lower frequencies 10% AEP to 2% AEP).

The uncertainty associated with the FFA ensemble is the lowest of the three components, but it interacts with the climate model uncertainty. Generally, the FFA uncertainty increases with the frequency, because the FFA is is sensitive to the slope of the flood frequency curve above the 5% AEP, which is fitted to a low sample size and extrapolated beyond the 40-year record length to the 1% AEP^34,35^. In the baseline, the FFA variance increases with the event frequency from 8% (10% AEP) to 11% (1% AEP). For the projections, the FFA variance patterns vary between the scenarios, whereas the 1% AEP variance decreases to 8–9% for SSP585, and increases to 12% (near-future) and 15% (far-future) for SSP245. Generally, the variance of component interactions is less than 3%, but in the case of SSP245 (far-future), the interaction effect of climate and FFA ensembles is 7% (p-value < 0.1).

**Fig. 9:**
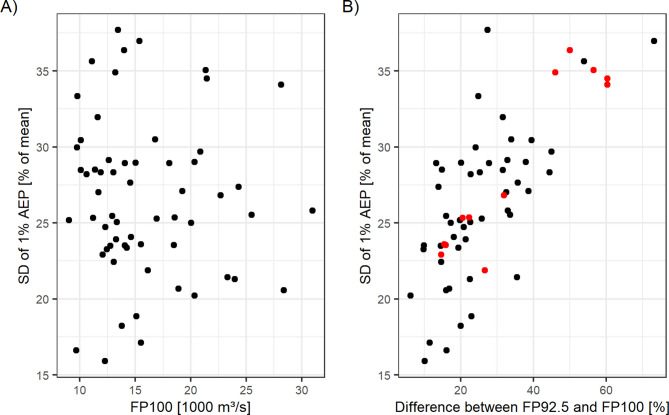
Comparison of the standard deviation of the 1% AEP event and the FP_100_ discharge (**A**). (**B**) compares the standard deviation of the 1% AEP event with the relative difference between the FP_100_ and FP_92.5_ discharges. The red points are the samples of the SSP245 scenario for the far-future. The standard deviation of the 1% AEP is affected by the relative difference between the FP_100_ and FP_92.5_ but there is no significant relationship between standard deviation of the 1% AEP and the FP_100_ discharge.

We attribute this CM-FFA interaction effect, and the higher FFA variance to the flood discharge time series projected for individual climate members. The Flood Percentile (FP_n_) aggregates the flood events by the n^th^ frequency percentile (see Supplementary Information 1). In the case of the far-future of SSP245, 5 out of 12 models predict > 40% higher FP_100_ discharge (highest discharge in the record) relative to the FP_92.5_ discharge (4th highest discharge) (Fig. 9). This large difference between the rarest and more frequent events increases the sampling uncertainty because the rarest events are removed or duplicated in the bootstrapping process, which increases the difference between the slopes of the individual flood frequency curves. The case of the far-future of SSP245 indicates that the FFA uncertainty is affected by projections of rare extreme precipitation events and is thus sensitive towards the selection of climate models and the time frame in which the most extreme events may or may not occur.

## Discussion

Current climate projections indicate with high confidence that heavy precipitation and pluvial floods increase with increasing Greenhouse Gas (GHG) emissions in the Central Himalayas, but are less certain how these increases affect fluvial floods^4,6^. Previous studies suggest an increase in fluvial flood discharges, and while direct comparisons are hampered by differences in the research design (e.g. spatial and temporal extent, datasets, models, catchments, event frequencies), our projections fall within the reported range of + 20 to + 108%^9,10,12,13^. We can attribute these changes to increasing rainfall-runoff, consistently predicted for a large ensemble of climate projections and hydrological model configurations, suggesting that projected increases in heavy precipitation will cause an intensification of river floods in the foreland of Central Himalayan catchments.

The uncertainty in predicting the hydrological response to climate change can exceed the uncertainty in predicting climate response to GHG emissions in mountainous catchments in which melt processes are important^36^. In the Himalayas, the uncertainty in hydrological modelling applications is increased by the coarse resolution of physical and climatic datasets and a lack of hydro-meteorological observations at higher elevations (> 4,000 masl)^5,23,29,37^. Here, the hydrological modelling is the largest source of uncertainty in the baseline. However, the ‘good’ to ‘very good’ modelling efficiencies^28^, the agreement in the seasonality of the runoff composition with other studies^11,21,22^, and the similarities in the projected changes in the hydrological behaviour, such as an earlier onset of the snow-melt season^11,38,39^, indicate that the hydrological ensemble reflects the catchment hydrology well. The relative uncertainty in the hydrological predictions decrease for future climates, which may be attributed to the dominance of rainfall-runoff in floodwater generation, as the rainfall-to-runoff conversion is less complex than other processes that include freezing, melting, and percolation.

This study does not consider projected land use changes in the experimental design, although the land use is an important component of the hydrological cycle and affects flood hazard characteristics^40^. In the case of Nepal, two drivers of land use change may be of particular relevance: (i) climatic changes shift climate zones and, thus, the natural vegetation cover^41,42^, and; (ii) the expansion or abandonment of agricultural terraces (e.g. caused by migration), which affect the surface and subsurface runoff generation and the hydrological connectivity and, thus, alter the contribution to the flood discharge^43,44^.

The Himalayan foreland in Nepal is, under both past and current climatic conditions, prone to flooding, with high mortalities and impacted populations, economic damages, and other repercussions, including increasing food insecurity and the outbreak of epidemics^1,45–47^. Future GHG emissions are likely to exacerbate these impacts. For the near-future, the relationship between flood magnitude and emissions is less pronounced due to the small differences between the SSP245 and SSP585 flood magnitudes from the relatively small differences in GHG emissions between the two climate scenarios (also possibly due to internal variability (i.e. natural climatic fluctuations) obscuring responses^48,49^. For the far-future however, our simulations suggest that GHG emissions cause a sustained flood hazard intensification - even for the SSP245 scenario, where emissions are higher for each year of the near-future than for the far-future, while the 30-year mean flood discharge continuously increases until the year 2099. The flood projections imply that: (i) Central Himalayan floods will intensify with time and emissions; (ii) this intensification is likely to continue for decades after the peak emissions and; (iii) flood magnitudes are likely to remain above current levels until the end of the century.

Flood magnitudes in the far-future are characterised by an increased spread of flood magnitude predictions between the individual CMIP6 members, ranging from + 9% to + 69% (SSP245) and − 12% to + 144% (SSP585) for the median 1%-AEP projections from the hydrological model and FFA ensemble. Furthermore, the FFA projections are sensitive to the slope of the frequency curves, and thus to the rarest, most extreme events (i.e., the FP_100_) and the discharge difference between the rarest and more common events (i.e., the FP_92.5_ and FP_100_). Both the climate model differences and the FFA sensitivity to rare events make the projection of design floods (i.e. the 1%-AEP event) susceptible to the unknown true frequency of the rarest projected events. Therefore, it is important to maintain a large ensemble of climate models to guarantee a larger sample size of the rarest events (i.e. the FP_100_ events of the climate models). If necessary, a new selection criterion should be developed for sub-setting climate models, which account for the frequency of extremes rather than the commonly used temperature and annual precipitation subset (e.g. dry-cold, dry-warm, wet-cold, wet-warm). Furthermore, a subset should include a combination of AOGCMs and ESMs, as these tend to project different rates of change.

The near-future flood projections indicate that the Karnali River is already in a phase of flood hazard intensification, so existing flood risk management strategies need to be adapted to maintain current flood protection levels. Flood defences, such as embankments and outlet structures, are typically designed and implemented on decadal time scales and, therefore, new projects should take the projected future changes in flood hazards into consideration^50,51^. The inherent uncertainties of future emissions and their impact on the climate and flood response create conflicts between designing protection levels for the worst-case scenario *versus* the economic feasibility of such structures. New, flexible, and anticipatory flood risk management strategies are therefore needed that can account for emerging new knowledge and understanding. Such strategies could include flexible designs for future flood risk infrastructure, which include the potential for later adaptations and upgrades, such as increases in storage space for floodwater, or increased dam walls levels. Additional strategies could include allocating space now for flood water retention areas within urban-zoning development plans, and policies and institutional workflows that reduce the period between planning and operation.

## Conclusions

This study presents an environmental modelling framework that uses high-resolution, large ensemble modelling to predict future design floods at the catchment scale for the Central Himalayan Region. We provide design floods (i.e. 1%-AEP) for future climate change scenarios, and the hydrological processes driving these potential changes, thereby providing practical information for climate change adaptation for flood risk management. The framework extends current uncertainty analyses by incorporating sampling uncertainty into the FFA. The uncertainty analysis highlights the sensitivity of the FFA to the flood discharge projections of individual climate models, emphasising the necessity to maintain large climate ensembles for flood impact analyses. These ensembles should contain both AOGCMs and ESMs, as these families differ in their flood projections.

The application of this framework projects the intensification of flood hazards over time and with increasing emissions. The hydrological and climate ensemble consistently predicts that the flood hazard intensification is driven by rainfall-runoff increases, which increases the evidence that increases in heavy precipitation will lead to increased river floods in the foreland of Central Himalayan catchments.

## Online methods

### Research design

We combine hydrological and statistical modelling with probabilistic climate projections to predict the potential changes in future flood hazards in the Karnali River in Nepal and China. In the first stage, we implement the hydrological model Spatial Processes in Hydrology (SPHY) for historical conditions (past and observed hydro-meteorological conditions) and identify an ensemble of behavioural parameter sets. This ensemble is then forced with probabilistic climate projections for three scenarios: the Baseline (1975–2014), the medium-emission scenario SSP245 (2020–2099) and the high-emission scenario SSP585 (2020–2099). In the last stage, a Flood Frequency Analysis (FFA) is conducted with the simulated flood discharges, which are classified by the Annual Maximum flow (AMAX). We compare the differences between the Baseline and the projected scenarios (SSP245 and SSP585) to quantify the changes in flood hazard magnitude and frequency between the past (1975–2014) and the near-future (2020–2059), as well as the far-future (2060–2099). We chose 40-year time frames over the commonly used 30-year time frames to increase the length of the flood record, which is the main source of uncertainty in the FFA^34^.

### Data processing

The utilised datasets comprise gridded and point-based datasets that determine the environmental (static) and climatic (dynamic) boundary conditions, plus calibration and validation datasets for the hydrological model (Supplementary Table 1). All gridded datasets are resampled to the modelling resolution (500 × 500 m) and reprojected to the UTM44N coordinate system (EPSG: 32644). The preprocessing of hydro-climatic data includes:

- Precipitation (historical): The network of precipitation gauges is concentrated in the floodplains and valleys, and precipitation at high elevations is poorly observed^29^. We therefore use monthly gridded satellite-based precipitation estimates (GPM IMERG Final Precipitation L3 1 Month V006^52^) and disaggregate them to the daily resolution using precipitation measurements of the Department of Hydrology and Meteorology, Nepal (DHM), following the approach of Arias-Hidalgo et al. (2013)^53^.
- Temperature (historical): We use the WATCH forcing Data methodology applied to ERA-Interim dataset (WFDEI)^54,55^ to determine the historical temperature boundary conditions. This dataset has a spatial resolution of 0.5 × 0.5° and is downscaled to the modelling resolution using a lapse rate derived from DHM temperature observations.
- Climate projections: We use the temperature and precipitation predictions of an ensemble of 12 CMIP6 Global Circulation Models (GCM), which have been bias-corrected using Empirical Quantile Mapping and downscaled to 0.25 × 0.25º by Mishra et al. (2020)^15^. The CanESM5 member was removed from the ensemble because it does not depict the monsoon seasonality in the catchment (Supplementary Fig. 3). The temperature data is downscaled to the modelling resolution using a lapse rate, and the precipitation data is downscaled using bilinear interpolation.
- Discharge: We use daily discharge data and stage-discharge observations (DHM) at the mountain outlet for the model calibration. The uncertainty in the discharge is estimated using the BaRatin approach^56^.

### The hydrological model

The catchment hydrology is simulated by the Spatial Processes in Hydrology (SPHY) model version 3.0^18^. SPHY is a distributed (raster-based) model that simulates soil processes, snow processes and evapotranspiration on the grid scale, and glacial processes on a sub-grid scale. The model distinguishes four types of runoff; rainfall-runoff Q_RR_, baseflow Q_BF_, snowmelt Q_SM_, and glacier melt Q_GM_ which compose the total runoff Q_TOT_ of each cell:

1 $$\:{Q}_{TOT}=\:{Q}_{RR}+\:{Q}_{BF}+{Q}_{SM}+{Q}_{GM}\:$$

The total runoff is then routed to the downstream cell based on a flow direction raster. The model implements a flow recession coefficient to account for channel friction (Supplementary Table 2).

The soil processes are calculated as in the SWAT and SWAP models^57^. The soil is divided into a rootzone layer SW_1_, a subzone layer SW_2_ and the groundwater layer SW_3_. The upper two layers SW_1_ and SW_2_ contribute to rainfall-runoff whereas the model simulates surface runoff (saturation excess runoff and infiltration excess runoff), and slower lateral flow from the soil layers. Percolation to the groundwater storage and baseflow are calculated using a recession coefficient to relate the baseflow to the groundwater recharge.

The reference evapotranspiration is estimated from the modified Hargreaves evapotranspiration equation^58^ and crop coefficients are used to estimate the potential evapotranspiration rates for different land covers^59^. Snow processes are simulated by a dynamic snow storage model^60^ using a degree-day-factor approach to quantify the snow melt^61^. Glacier melt is also calculated using the degree-day factor approach whereas separate factors are defined for clean-ice and debris-covered glaciers. The evolution of glaciers (the retreat or advance) is simulated from the mass balance (snow accumulation – glacier melt) using a mass conserving ice redistribution approach which is applied once per year^19^.

### Hydrological modelling setup

We use the SPHY model version 3.0 with the dynamic glaciers, infiltration, groundwater and snow modules. The model is applied at 500 × 500 m grid resolution with daily time steps. It is calibrated and validated for 2002–2006 and 2007–2015, respectively.

A stepwise calibration approach is used to identify behavioural parameter sets. In the first stage, a Regional Sensitivity Analysis with 1,500 Latin Hypercube Samples (LHS)^62^ of 21 parameters is conducted to identify sensitive parameters. In the second stage, the 15 sensitive parameters are calibrated within a GLUE framework using 10,000 parameter sets sampled with LHS. We implemented two scaling factors as calibration parameters: (i) a precipitation correction factor to account for the bias in the precipitation data (similar to Lutz et al. (2014)^11^, and (ii) a multiplication factor for the crop coefficients which control the evapotranspiration for different land cover classes (similar to Khanal et al. (2021)^19^. The precipitation correction factor is used for the model calibration but is not transferred to the climate projections because the CMIP6 data is bias-corrected.

We focus on the model performance during high flows for selecting the behavioural parameter sets using a multi-criteria approach to increase the robustness of the ensemble predictions^63^. However, we exclude parameter sets with percentage BIAS (PBIAS) exceeding ± 30% for the annual actual evapotranspiration from MODIS, the 8-day snow extent from MODIS, and the discharge during the winter months to prevent the selection of parameter sets that do not depict critical aspects of the catchment hydrological behaviour. Of the remaining parameter sets, we select the 25 members with the highest Nash-Sutcliffe Efficiency (NSE), PBIAS of high flows (≥ 5,000 m^3^/s), and a modified version of the extended GLUE (eGLUE)^64^ to account for the uncertainty in the discharge observations. The eGLUE quantifies the number of days during which the simulated discharge is within the uncertainty interval of the observed discharge. The modified eGLUE is the sum of the simulated discharge for all days that fall within the observed confidence interval divided by the total number of days. This adjustment is made to prevent lower flows, which occur throughout most of the year, from dominating the classification. After the removal of duplicates, 64 parameter sets are maintained.

### Flood frequency analysis

We apply a Flood Frequency Analysis (FFA) to quantify the changes in flood magnitudes for the projected climate scenarios. The Flood Frequency Analysis (FFA) is a statistical method that utilises an Extreme Value Distribution (EVD) to estimate flood magnitude based on flood frequency (occurrence probability) from a record of flood events^65^. The FFA is fitted to each AMAX record of the modelling ensemble (12 CMIP6 members X 64 hydrological models) using the L-Moments approach for parameter estimation^66^. We evaluated the performance of eight EVD because it is generally impossible to determine the most suitable distribution before the application^65^, and selected the Wakeby distribution for the Flood frequency analysis (Supplementary Table 4). We employ a bootstrapping approach (*n* = 1,000) based on Burn (2003)^17^ to estimate the sampling uncertainty, which relates to the difference between the distribution used in the FFA and the true distribution of the flood discharge. The flood discharges are sorted before the bootstrapping, and the same bootstrap samples are taken for all combination of climate models and hydrological parametersets to ensure the comparability of FFA samples in the uncertainty decomposition.

## Supplementary Information

Below is the link to the electronic supplementary material.

Supplementary Material 1

## Data Availability

The code and datasets to reproduce the figures are available on Zenodo at 10.5281/zenodo.16408489.

## References

[1] EM-DAT/CRED EM-DAT: The emergency events database. (2021).

[2] The World Bank Group & Asian Development Bank. *Climate Risk Country Profile: Nepal* https://climateknowledgeportal.worldbank.org/sites/default/files/2021-05/15720-WB_Nepal%20Country%20Profile-WEB.pdf (2021).

[3] Ahmed, M. *Assessing the Costs of Climate Change and Adaptation in South Asia* (Asian Development Bank, 2014).

[4] Gulev, S. K. et al. Changing State of the Climate System. *Climate Change 2021: The Physical Science Basis. Contribution of Working Group I to the Sixth Assessment Report of the Intergovernmental Panel on Climate Change* 287–422 10.1017/9781009157896.004 (2023).

[5] Scott, C. A. et al. Water in the Hindu Kush himalaya. in The Hindu Kush Himalaya Assessment: Mountains, Climate Change, Sustainability and People (eds (eds Wester, P., Mishra, A., Mukherji, A. & Shrestha, A. B.) 257–299 (Springer International Publishing, Cham, doi:10.1007/978-3-319-92288-1_8. (2019).

[6] Seneviratne, S. I. et al. Weather and climate extreme events in a changing climate. *Climate Change 2021: The Physical Science Basis. Contribution of Working Group I to the Sixth Assessment Report of the Intergovernmental Panel on Climate Change* 1513–1766 10.1017/9781009157896.013 (2023).

[7] Zhang, S. et al. Reconciling disagreement on global river flood changes in a warming climate. *Nat. Clim. Change*. **12**, 1160–1167 (2022).

[8] Hirabayashi, Y. et al. Global flood risk under climate change. *Nat. Clim. Change*. **3**, 816–821 (2013).

[9] Hirabayashi, Y., Tanoue, M., Sasaki, O., Zhou, X. & Yamazaki, D. Global exposure to flooding from the new CMIP6 climate model projections. *Sci. Rep.* **11**, 3740 (2021). 33580166 10.1038/s41598-021-83279-w PMC7881105

[10] Dankers, R. et al. First look at changes in flood hazard in the Inter-Sectoral impact model intercomparison project ensemble. *Proc. Natl. Acad. Sci.* **111**, 3257–3261 (2014). 24344290 10.1073/pnas.1302078110 PMC3948307

[11] Lutz, A. F., Immerzeel, W. W., Shrestha, A. B. & Bierkens, M. F. P. Consistent increase in high asia’s runoff due to increasing glacier melt and precipitation. *Nat. Clim. Change*. **4**, 587–592 (2014).

[12] Pechlivanidis, I. G. et al. Analysis of hydrological extremes at different hydro-climatic regimes under present and future conditions. *Clim. Change*. **141**, 467–481 (2017).

[13] Wijngaard, R. R. et al. Future changes in hydro-climatic extremes in the upper Indus, Ganges, and Brahmaputra river basins. *PLOS One*. **12**, e0190224 (2017). 29287098 10.1371/journal.pone.0190224 PMC5747487

[14] Pappenberger, F. & Beven, K. J. Ignorance is bliss: or seven reasons not to use uncertainty analysis. *Water Resour. Res.* **42**, W05302 (2006).

[15] Mishra, V., Bhatia, U. & Tiwari, A. D. Bias-corrected climate projections for South Asia from coupled model intercomparison Project-6. *Sci. Data*. **7**, 338 (2020). 33046709 10.1038/s41597-020-00681-1 PMC7550601

[16] Beven, K. & Binley, A. The future of distributed models: model calibration and uncertainty prediction. *Hydrol. Process.* **6**, 279–298 (1992).

[17] Burn, D. H. The use of resampling for estimating confidence intervals for single site and pooled frequency analysis / Utilisation d’un rééchantillonnage pour l’estimation des intervalles de confiance Lors d’analyses fréquentielles mono et multi-site. *Hydrol. Sci. J.* **48**, 25–38 (2003).

[18] Terink, W., Lutz, A. F., Simons, G. W. H., Immerzeel, W. W. & Droogers, P. SPHY v2.0: Spatial processes in hydrology. *Geosci. Model Dev.* **8**, 2009–2034 (2015).

[19] Khanal, S. et al. Variable 21st century climate change response for rivers in high mountain Asia at seasonal to decadal time scales. *Water Resour. Res.* **57**, e2020WR029266 (2021).

[20] Kuma, P., Bender, F. A. & Jönsson, A. R. Climate model code genealogy and its relation to climate feedbacks and sensitivity. *J. Adv. Model. Earth Syst.* **15**, e2022MS003588 (2023).

[21] Bookhagen, B. & Burbank, D. W. Toward a complete Himalayan hydrological budget: Spatiotemporal distribution of snowmelt and rainfall and their impact on river discharge. *J. Geophys. Research: Earth Surf.* **115**, F03019 (2010).

[22] Andermann, C., Crave, A., Gloaguen, R., Davy, P. & Bonnet, S. Connecting source and transport: suspended sediments in the Nepal Himalayas. *Earth Planet. Sci. Lett.* **351**, 158–170 (2012).

[23] Nepal, S., Krause, P., Flügel, W. A., Fink, M. & Fischer, C. Understanding the hydrological system dynamics of a glaciated alpine catchment in the Himalayan region using the J2000 hydrological model. *Hydrol. Process.* **28**, 1329–1344 (2014).

[24] Bhattarai, S., Zhou, Y., Shakya, N. M. & Zhao, C. Hydrological modelling and climate change impact assessment using HBV light model: a case study of Narayani river Basin, Nepal. *Nat. Environ. Pollution Technol.* **17**, 691–702 (2018).

[25] Dahal, P., Shrestha, M. L., Panthi, J. & Pradhananga, D. Modeling the future impacts of climate change on water availability in the Karnali river basin of Nepal himalaya. *Environ. Res.* **185**, 109430 (2020). 32247907 10.1016/j.envres.2020.109430

[26] Pandey, V. P., Dhaubanjar, S., Bharati, L. & Thapa, B. R. Spatio-temporal distribution of water availability in Karnali-Mohana Basin, Western nepal: hydrological model development using multi-site calibration approach (Part-A). *J. Hydrology: Reg. Stud.* **29**, 100690 (2020).

[27] Pradhananga, S., Nepal, S., Kamal, S. K. & Hafeez, M. Climate change will exacerbate seasonal flow variability in the Karnali river basin: implications for water, energy, and agriculture sectors. *Hydrol. Res.* **56**, 471–491 (2025).

[28] Moriasi, D. N., Gitau, M. W., Pai, N. & Daggupati, P. Hydrologic and water quality models: performance measures and evaluation criteria. *Trans. ASABE*. **58**, 1763–1785 (2015).

[29] Immerzeel, W. W., Wanders, N., Lutz, A. F., Shea, J. M. & Bierkens, M. F. P. Reconciling high-altitude precipitation in the upper indus basin with glacier mass balances and runoff. *Hydrol. Earth Syst. Sci.* **19**, 4673–4687 (2015).

[30] Immerzeel, W. W., Pellicciotti, F. & Shrestha, A. B. Glaciers as a proxy to quantify the Spatial distribution of precipitation in the Hunza basin. *Mt. Res. Dev.* **32**, 30–38 (2012).

[31] Dhami, B., Himanshu, S. K., Pandey, A. & Gautam, A. K. Evaluation of the SWAT model for water balance study of a mountainous snowfed river basin of Nepal. *Environ. Earth Sci.* **77**, 21 (2018).

[32] Shrestha, A. & Thakuri, P. S. Spatial and Temporal variations in water balance components of a Class-II river system in nepal: a SWAT-based analysis. *H2Open J.* **8**, 42–58 (2025).

[33] Zhang, S., Zhou, Z., Peng, P. & Xu, C. A new framework for estimating and decomposing the uncertainty of climate projections. *J. Clim.* **37**, 365–384 (2024).

[34] Apel, H., Merz, B. & Thieken, A. H. Quantification of uncertainties in flood risk assessments. *Int. J. River Basin Manage.* **6**, 149–162 (2008).

[35] Kjeldsen, T. R., Lamb, R. & Blazkova, S. Uncertainty in flood frequency analysis. In *Applied Uncertainty Analysis for Flood Risk Management* (eds Beven, K. & Hall, J.) 153–197 (Imperial College Press, 2014).

[36] Giuntoli, I., Vidal, J. P., Prudhomme, C. & Hannah, D. M. Future hydrological extremes: the uncertainty from multiple global climate and global hydrological models. *Earth Sys. Dyn.* **6**, 267–285 (2015).

[37] Collier, E. & Immerzeel, W. W. High-resolution modeling of atmospheric dynamics in the Nepalese himalaya. *J. Geophys. Research: Atmos.* **120**, 9882–9896 (2015).

[38] Caretta, A. M. M. A., Arfanuzzaman, R. B. M., Morgan, S. M. R. & Kumar, M. Water. Climate change 2022: Impacts, adaptation, and vulnerability. In *Contribution of Working Group II to the Sixth Assessment Report of the Intergovernmental Panel on Climate Change*. (2022).

[39] Hock, R. et al. High mountain areas. In *IPCC special report on the ocean and cryosphere in a changing climate* 131–202 (eds H. O. Pörtner, DC Roberts, V. Masson-Delmotte, P. Zhai, M. Tignor, E).

[40] Rogger, M. et al. Land use change impacts on floods at the catchment scale: challenges and opportunities for future research. *Water Resour. Res.* **53**, 5209–5219 (2017). 28919651 10.1002/2017WR020723 PMC5575485

[41] Beck, H. E. et al. High-resolution (1 km) Köppen-Geiger maps for 1901–2099 based on constrained CMIP6 projections. *Sci. Data*. **10**, 724 (2023). 37872197 10.1038/s41597-023-02549-6 PMC10593765

[42] Malla, R., Neupane, P. R. & Köhl, M. Climate change impacts: vegetation shift of broad-leaved and coniferous forests. *Trees Forests People*. **14**, 100457 (2023).

[43] Gardner, R. A. M. & Gerrard, A. J. Runoff and soil erosion on cultivated rainfed terraces in the middle hills of Nepal. *Appl. Geogr.* **23**, 23–45 (2003).

[44] Arnáez, J., Lana-Renault, N., Lasanta, T., Ruiz-Flaño, P. & Castroviejo, J. Effects of farming terraces on hydrological and Geomorphological processes. A review. *CATENA* **128**, 122–134 (2015).

[45] DWIDP. Annual disaster review 2014. (2015).

[46] Hannah, D. M. et al. Water and sanitation for all in a pandemic. *Nat. Sustain.* **3**, 773–775 (2020).

[47] MoHA. Nepal Disaster Report: The hazardscape and vulnerability. (2009).

[48] Hawkins, E. & Sutton, R. The potential to narrow uncertainty in regional climate predictions. *Bull. Am. Meteorol. Soc.* **90**, 1095–1108 (2009).

[49] Wu, Y. et al. Quantifying the uncertainty sources of future climate projections and narrowing uncertainties with bias correction techniques. *Earth’s Future*. **10**, e2022EF002963 (2022).

[50] Sinha, R. & Kosi Rising Waters, dynamic channels and human disasters. *Economic Political Wkly.* **43**, 42–46 (2008).

[51] Dinesh Kumar Mishra. The Bihar flood story. *Economic Political Wkly.* **32**, 2206–2217 (1997).

[52] Huffman, G. et al. J. NASA global precipitation measurement (GPM) - integrated multi-satellitE retrievals for GPM (IMERG). **38** (2019).

[53] Arias-Hidalgo, M., Bhattacharya, B., Mynett, A. & Van Griensven, A. Experiences in using the TMPA-3B42R satellite data to complement rain gauge measurements in the Ecuadorian coastal foothills. *Hydrol. Earth Syst. Sci.* **17**, 2905–2915 (2013).

[54] Weedon, G. et al. Creation of the WATCH forcing data and its use to assess global and regional reference crop evaporation over land during the twentieth century. *J. Hydrometeorol.* **12**, 823–848 (2011).

[55] Weedon, G. P. et al. The WFDEI meteorological forcing data set: WATCH forcing data methodology applied to ERA-Interim reanalysis data. *Water Resour. Res.* **50**, 7505–7514 (2014).

[56] Le Coz, J., Renard, B., Bonnifait, L., Branger, F. & Le Boursicaud, R. Combining hydraulic knowledge and uncertain gaugings in the Estimation of hydrometric rating curves: A bayesian approach. *J. Hydrol.* **509**, 573–587 (2014).

[57] Neitsch, S. L., Arnold, J. G., Kiniry, J. R. & Williams, J. R. *Soil and Water Assessment Tool Theoretical Documentation Version 2009*. (2011).

[58] Hargreaves, G. H. & Samani, Z. A. Reference crop evapotranspiration from temperature. *Appl. Eng. Agric.* **1**, 96–99 (1985).

[59] Allen, R. G., Pereira, L. S., Raes, D. & Smith, M. Crop evapotranspiration-Guidelines for computing crop water requirements-FAO irrigation and drainage paper 56. *Fao Rome*. **300**, D05109 (1998).

[60] Kokkonen, T., Koivusalo, H., Jakeman, T. & Norton, J. Construction of a degree-day snow model in the light of the ten iterative steps in model development. (2006).

[61] Hock, R. Temperature index melt modelling in mountain areas. *J. Hydrol.* **282**, 104–115 (2003).

[62] McKay, M. D., Beckman, R. J. & Conover, W. J. A comparison of three methods for selecting values of input variables in the analysis of output from a computer code. *Technometrics* **21**, 239–245 (1979).

[63] Legates, D. R. & McCabe, G. J. Evaluating the use of goodness-of-fit measures in hydrologic and hydroclimatic model validation. *Water Resour. Res.* **35**, 233–241 (1999).

[64] Liu, Y., Freer, J., Beven, K. & Matgen, P. Towards a limits of acceptability approach to the calibration of hydrological models: extending observation error. *J. Hydrol.* **367**, 93–103 (2009).

[65] Hosking, J. R. M. & Wallis, J. R. *Regional Frequency analysis: an Approach Based on L-Moments*10.1017/CBO9780511529443 (Cambridge University Press, 1997).

[66] Hosking, J. R. M. & L-Moments Analysis and Estimation of distributions using linear combinations of order statistics. *J. Royal Stat. Soc. Ser. B (Methodological)*. **52**, 105–124 (1990).

[67] Jarvis, A., Reuter, H., Nelson, A. & Guevara, E. Hole-filled SRTM for the globe Version 4. (2008).

