# Supplementary material for: Increased rainfall-runoff drives flood hazard intensification in Central Himalayan river systems
Source: Sci Rep. 2025 Dec 8;15:42277. doi: 10.1038/s41598-025-26815-2 (PMC12685963; doi:10.1038/s41598-025-26815-2)
Supplement: Supplementary file 1 — Supplementary Material 1 [file 41598_2025_26815_MOESM1_ESM.docx]

# **Supplements**

## **Section 1 – Datasets**

Table S 1: Overview of the datasets used in the hydrological modelling.

| Type | Variable | Dataset | Usage | Source |
| --- | --- | --- | --- | --- |
| Static | Topography | HydroSHED V 1.0 | Watershed delineation, temperature downscaling, Flow routing | Lehner et al. (2008)^1^ |
|  | Soil | HiHydroSoils | Soil saturated water content, field capacity, permanent wilting point of the root zone and subzone layers. Processed with the SPHY-Preprocessor^2^ | De Boer (2016)^3^ |
|  | Geology | Physiographic Regions of Nepal | Demarcation of zones with different geological properties | Uddin et al. (2015)^4^ |
|  | Vegetation | Globcover 2009 V2.3 | Land cover classification | Arino et al. (2012)^5^ |
|  | Glacier | Randolph Glacier Inventory 6.0 | Glacier delineation | RGI Consortium (2017)^6^ |
|  |  | Debris-covered glaciers | Classification into debris-covered and free-ice glaciers | Scherler et al. (2018)^7^ |
|  |  | Ice thickness | Glacier depth | Farinotti et al. (2019)^8^ |
| Climate | Temperature  (historical) | WFDEI | Minimum, maximum and mean daily air temperature | Weedon et al. (2011, 2014)^9,10^ |
|  | Precipitation (historical) | GPM IMERG Final Precipitation L3 1 Month V006 | Monthly precipitation maps | Huffman et al. (2019)^11^ |
|  |  | Gauged precipitation (daily) | Temporal disaggregation of monthly precipitation maps | Department of Hydrology and Meteorology, Nepal |
|  | Climate projections | Probabilistic climate models. | Temperature and precipitation of 12 downscaled and bias-corrected CMIP6 models. Three scenarios: Baseline, SSP245, SSP585 | Mishra et al. (2020)^12^ |
| Evalu-ation | Actual Evapo-transpiration | MODIS 16A3GF V006 | Model calibration | Running et al (2019)^13^ |
|  | Discharge | Gauged discharge  (daily) | Model calibration | Department of Hydrology and Meteorology, Nepal |
|  |  | DHM stage-discharge observations | Uncertainty estimation of discharge data | Department of Hydrology and Meteorology, Nepal |
|  | Snow extent | MOYDGL06 | 8-day snow cover composites used for model calibration | Muhammad and Thapa (2020)^14^ |


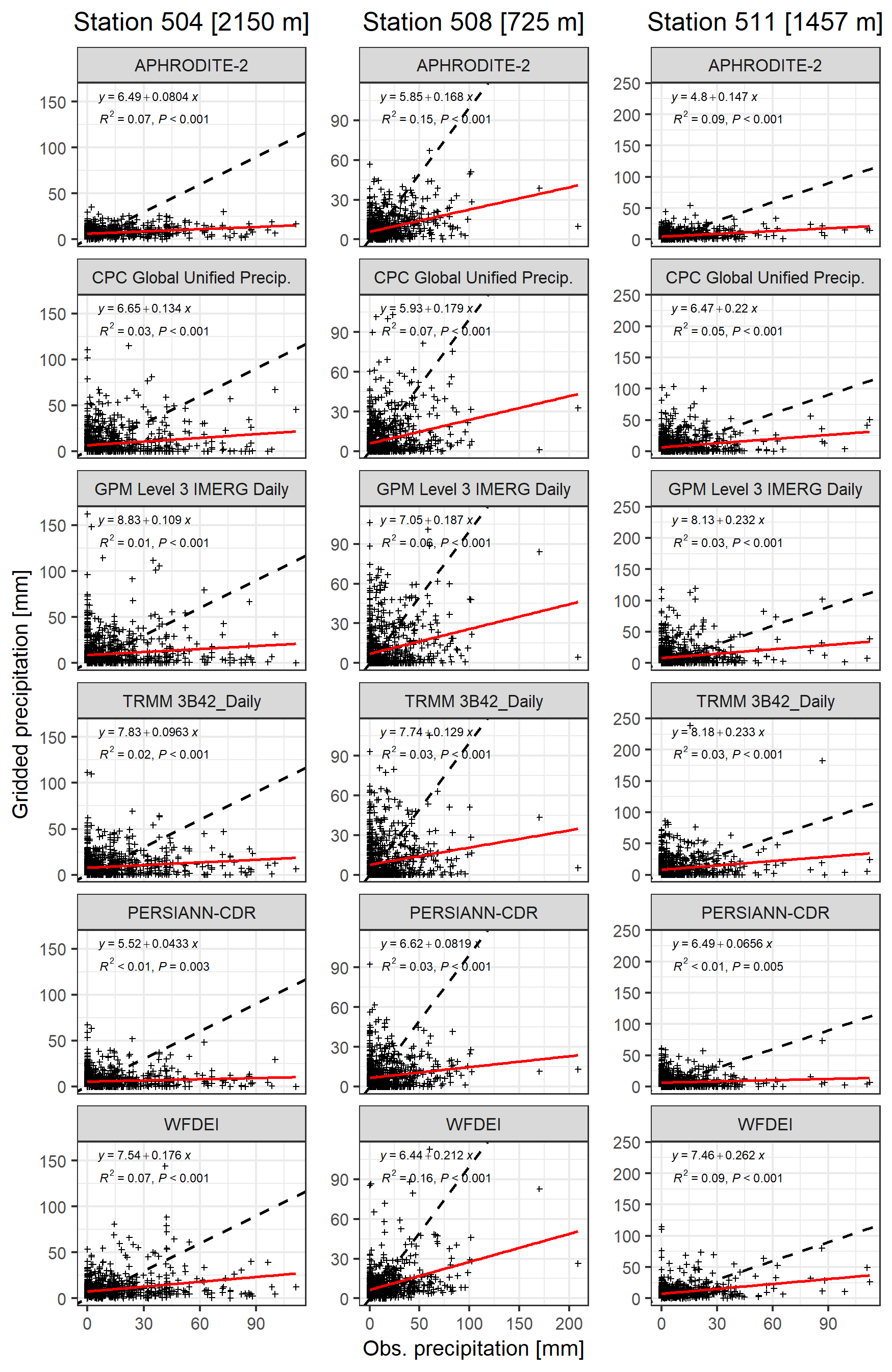


Figure S 1: Comparison of gridded daily precipitation products with precipitation gauges (DHM) in the Karnali River catchment (the station locations are presented in Fig. S 1). The investigated period is 2000 - 2009. The red line indicates the linear regression model, and the black dashed line indicates the line of the perfect fit. The low agreement of the precipitation estimates with the observations indicates the poor performance of daily gridded estimates, which hampers their application in hydrological modelling. We, therefore, used disaggregated monthly gridded precipitation (see Figure S 3).


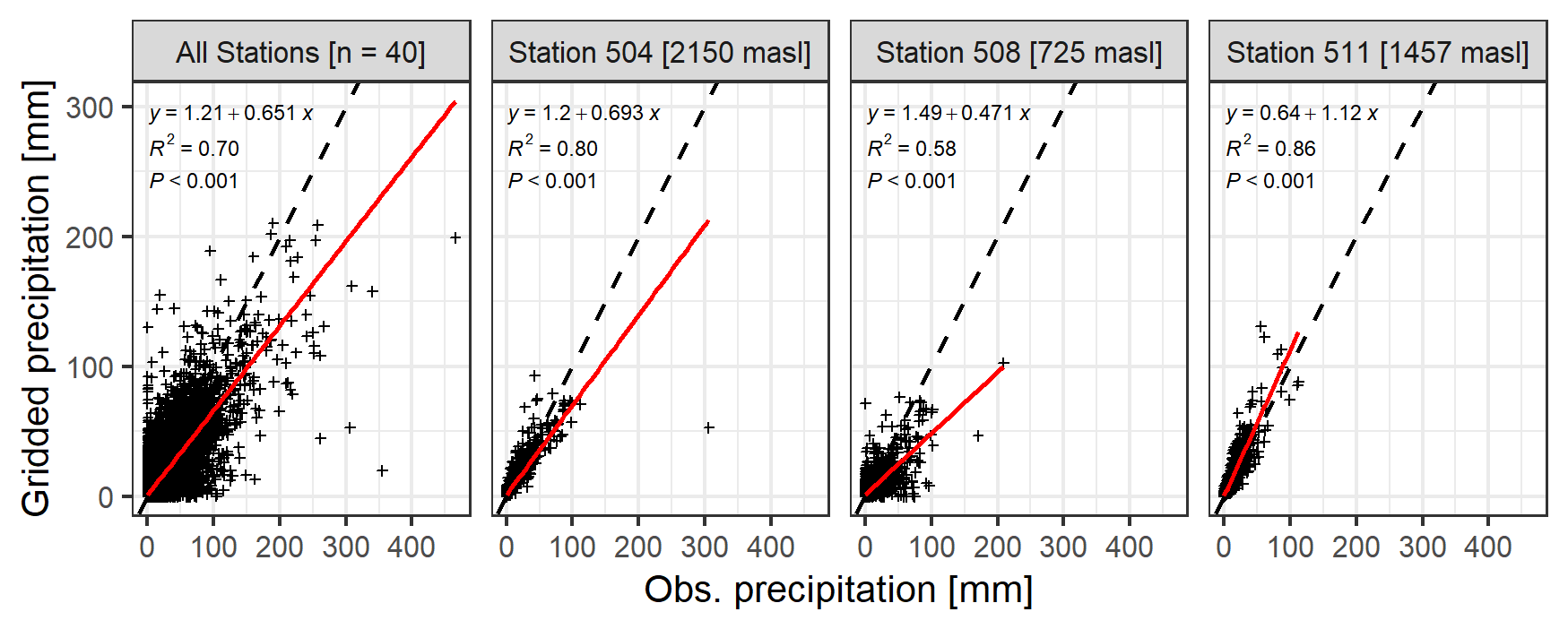


Figure S 2: Comparison of the disaggregated monthly GPM precipitation and the observed daily precipitation. The red lines indicate linear regression models, and the black dashed lines indicate the line of the perfect fit. The disaggregation improved the correlation between the gridded product and gauge observations, and the disaggregated dataset is used in the historical simulations (calibration and validation) of the hydrological model.


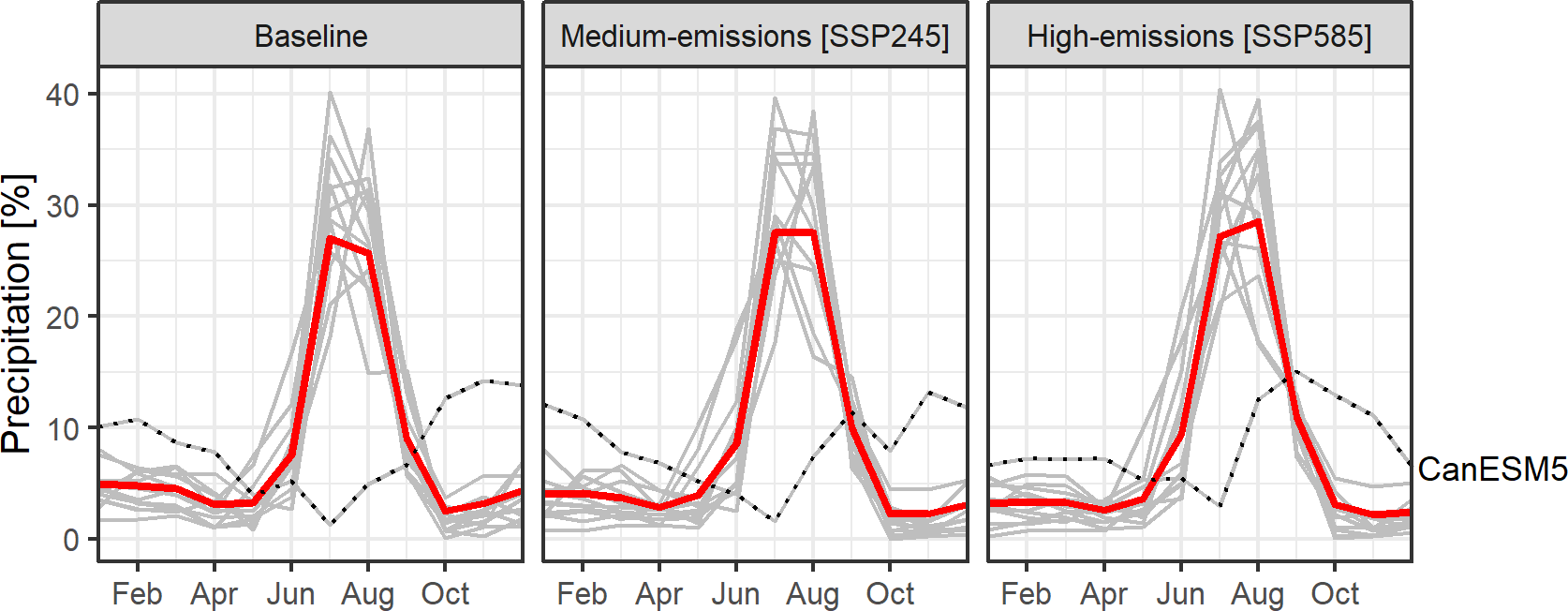


Figure S 3: Mean monthly precipitation of the 13 downscaled and bias-corrected CMIP6 members^12^ as a percentage of the annual precipitation for the baseline (1985 – 2014) and the projected scenarios (2070 – 2099). The red line represents the ensemble mean precipitation, and the grey lines denote the precipitation of the individual ensemble members. The dotted line represents the distribution of the CanESM5 model, which does not accurately capture monsoon seasonality (approx. 80% of annual precipitation occurs during the monsoon season) and is therefore not used in climate change modelling. The precipitation was averaged (mean) over the catchment.

## Section 2 – Environmental Modelling

Table S 2: The ranges of the sensitive parameters of the hydrological modelling of the Spatial Processes in HYdrology (SPHY) model (64 parameter sets).

| Module | Parameter | Description | Unit | Parameter value | | |
| --- | --- | --- | --- | --- | --- | --- |
|  |  |  |  | Min | Max | Median |
| Glacier | gDDF_cl | Degree-day factor debris-free glaciers | mm ºC^-1^ day^-1^ | 1.55 | 8.90 | 6.71 |
|  | gDDF_db | Degree-day factor debris-covered glaciers | mm ºC^-1^ day^-1^ | 1.80 | 8.94 | 6.89 |
|  | glacF | Fraction of glacier melt that becomes glacier runoff | - | 0.31 | 0.60 | 0.49 |
| Ground-water | deltaGW | Groundwater recharge delay time | days | 20 | 46 | 26 |
|  | alphaGW | Recession coefficient | day^-1^ | 0.02 | 0.04 | 0.03 |
|  | gwSat | Saturated soil water content of the groundwater layer | mm | 325 | 500 | 390 |
|  | h_gw | Initial groundwater table height | m | 1.10 | 2.10 | 1.60 |
| Routing | recessCoef | Flow recession coefficient | - | 0.75 | 0.95 | 0.84 |
| Snow | sDDF | Degree-day factor for snow | mm ºC^-1^ day^-1^ | 2.32 | 8.97 | 5.72 |
|  | Tcrit | Temperature threshold for precipitation to fall as snow | ºC | -3.5 | -0.6 | -2.4 |
| Soil | alphaInf | Fraction of daily precipitation that occurs during the hour of most intense rainfall | - | 0.16 | 0.40 | 0.34 |
|  | lambda | Infiltration coefficient that affects the speed of infiltration capacity reduction | - | 0.26 | 0.79 | 0.44 |
|  | kEff | Effective saturated hydraulic conductivity | - | 0.20 | 0.50 | 0.24 |
| Correction  factors | precipFactor | Precipitation correction factor | - | 1.35 | 1.42 | 1.40 |
|  | kcFactor | Crop coefficient multiplication factor | - | 0.80 | 1.19 | 0.85 |

Table S 3: Minimum, maximum and percentile (P_n_) efficiencies of the hydrological modelling ensemble for the calibration period (2002 – 2006) and validation period (2007-2015).

| Efficiency | Period | Min | P_10_ | P_50_ | P_90_ | Max |
| --- | --- | --- | --- | --- | --- | --- |
| NSE | Calibration | 0.75 | 0.78 | 0.85 | 0.86 | 0.87 |
|  | Validation | 0.71 | 0.743 | 0.82 | 0.84 | 0.85 |
| R2 | Calibration | 0.77 | 0.79 | 0.85 | 0.86 | 0.87 |
|  | Validation | 0.8 | 0.82 | 0.84 | 0.85 | 0.86 |
| PBIAS (≥5,000 m^3^/s) | Calibration | -32 | -31 | -25 | -18 | -15 |
|  | Validation | -27 | -24 | -16 | -5 | -1 |
| eGLUE_mod_ | Calibration | 318 | 419 | 533 | 582 | 601 |
|  | Validation | 408 | 470 | 540 | 600 | 621 |

Table S 4: Median efficiencies of Extreme Value Distributions (EVD) fitted for the flood discharges predicted by the hydrological ensemble (n =12 CMIP6 models x 64 hydrological models x 5 scenario and period combinations). The Wakeby distribution performs best and is selected as the EVD for the Flood Frequency Analysis.

| Extreme value distribution | Anderson-Darling Test statistic | Anderson-Darling Test P-Value | R^2^ | RMSE |
| --- | --- | --- | --- | --- |
| Wakeby | 0.205 | 0.977 | 0.992 | 0.028 |
| Generalized extreme value | 0.287 | 0.947 | 0.989 | 0.033 |
| Generalized logistic | 0.331 | 0.911 | 0.988 | 0.036 |
| Pearson Type III | 0.27 | 0.91 | 0.988 | 0.033 |
| Weibull | 0.247 | 0.839 | 0.988 | 0.034 |
| Gumbel | 0.414 | 0.833 | 0.984 | 0.041 |
| Exponential | 0.401 | ≤ 0.05 | 0.968 | 0.056 |
| Gen. pareto | 0.277 | ≤ 0.05 | 0.984 | 0.039 |


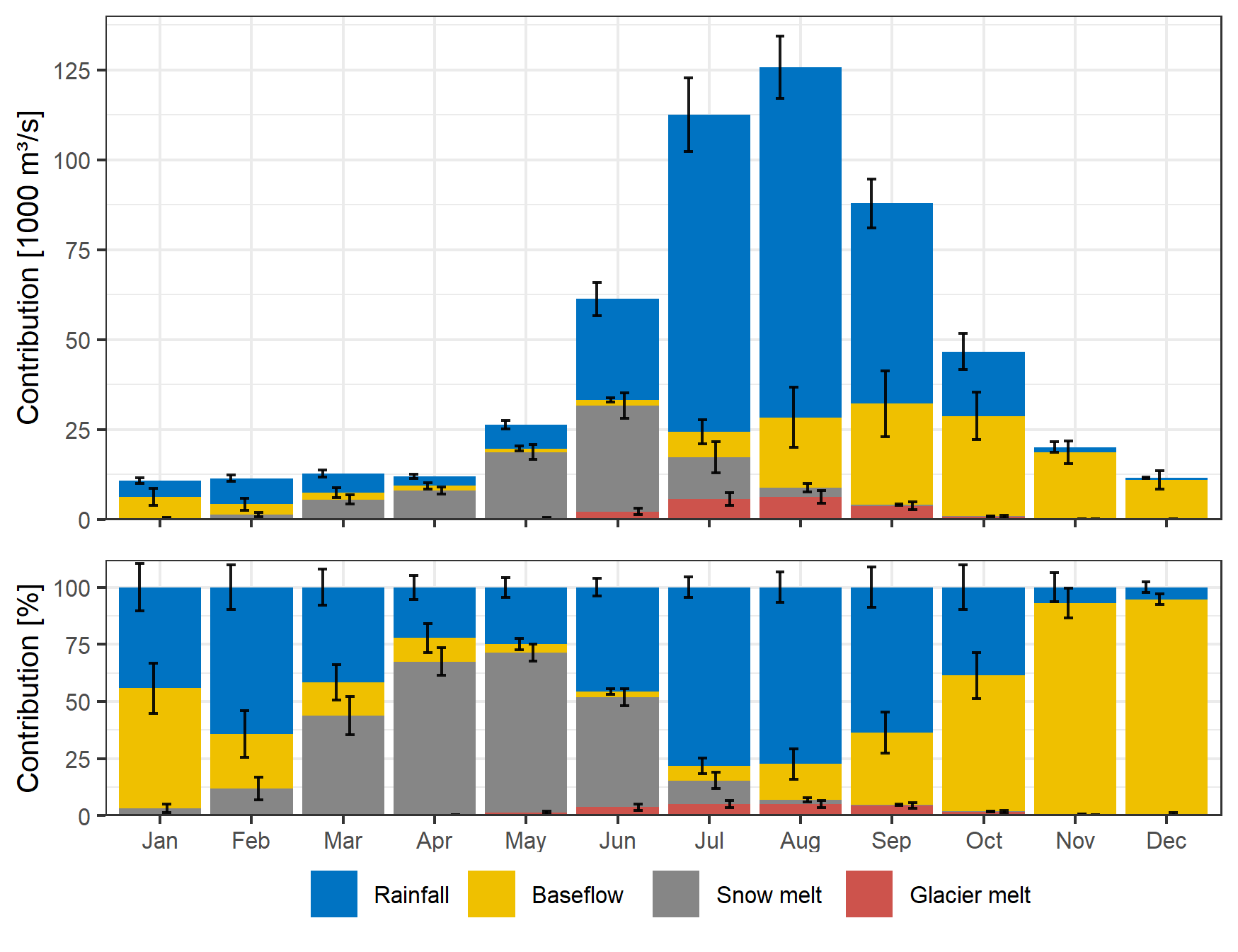


Figure S 4: Mean monthly runoff composition of the Karnali River. The error bars indicate the standard deviation originating from the hydrological parameterisation and differences between the year.

## Section 3 – Flood Projections

**Supplementary information 1: Flood percentile calculation**

The ensemble predictions of flood discharges (hydrological modelling) are aggregated by the flood percentile (FP) which describes the flood discharge of the i-th percentile where i is:

$i = \frac{\mathrm{rank}}{record length}*100$,

where the rank is the position of the flood discharge in ascending order. For example, FP_2.5_ is the discharge of the lowest predicted flood event and FP_100_ is the discharge of the highest flood event in the 40-year record. The flood percentiles provide a more meaningful measure to aggregate ensembles than a temporal aggregation. A temporal aggregation does not take the internal climate variability into account which would lead to the aggregation of smaller and larger events (i.e. the CMIP6 members predict large events in different years). Furthermore, the members of the hydrological ensemble may react differently to the climate input, and this information would be lost in the temporal aggregation. Instead, it is more meaningful to aggregate the flood predictions by frequency, which also enables the comparison with the predictions of the FFA.

For example, the FP_92.5_ is the 4^th^ highest flood prediction in the 40-year record ensemble member and is hence predicted statistically once every ten years and can be compared with the flood magnitude of the 1-in-10-year (10%-Annual Exceedance Probability (AEP)) event. The FP_97.5_ is the second-highest flood prediction and is hence predicted statistically once every twenty years and can be compared with the magnitude of the 1-in-20-year (5%-AEP) event.

**Supplementary information 2: Estimation of the uncertainty sources in the ensemble predictions**

The three components (climate modelling, hydrological modelling and the Flood Frequency Analysis) introduce uncertainty. The contribution of the sources is estimated by calculating the standard deviation for each component and averaging it over the other components.

Climate: We calculate the standard deviation across the 12 CMIP6 members for each of the 64 hydrological models and take the mean of these 64 values.

Hydrology: We calculate the standard deviation across the 64 hydrological models for each of the 12 CMIP6 members and take the mean of these 12 values.

Flood frequency analysis: We calculate the standard deviation of the 1,000 flood frequency curves for each combination of climate and hydrological models (12 x 64) and calculate the mean of these 768 values.

The mean prediction range provides information about the variability in each component’s predictions. However, it is worth noting that the ranges overlap between the components and that the total ensemble’s standard deviation is smaller than the sum of the component-wise standard deviations.


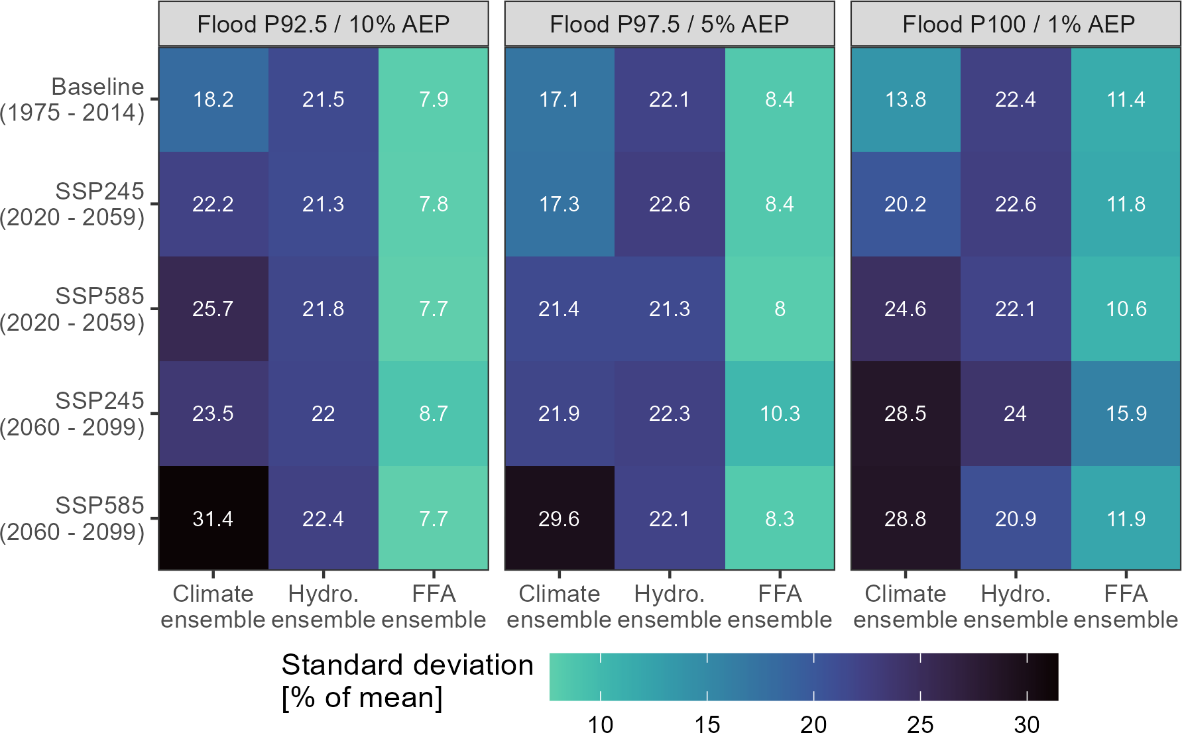


Figure S 5: The sources of uncertainty are indicated by the mean standard deviation (σ) of the ensemble predictions. We compare the Flood Percentiles (climate and hydrological ensembles) with the Annual Exceedance Probability (FFA ensemble). The σ of climate ensemble is averaged over the hydrological ensemble; the σ of the hydrological ensemble is averaged over the climate ensemble; the σ of the FFA ensemble is averaged over the climate and hydrological ensemble. Additional information about the calculation is provided in Supplementary Information 2.

Table S 5: P-values of the 3-Way ANOVA for the hydrological ensemble (HY), climate model ensemble (CM), Flood Frequency Ensemble (FFA), and component interactions.

| Scenario | Period | Return Period | HY | CM | FF | HY:CM | HY:FF | CM:FF |
| --- | --- | --- | --- | --- | --- | --- | --- | --- |
| Baseline | 1975-2014 | 10 | <0.01 | <0.01 | <0.01 | <0.01 | >0.1 | >0.1 |
| Baseline | 1975-2014 | 100 | <0.01 | <0.01 | <0.01 | <0.01 | >0.1 | >0.1 |
| SSP245 | 2020-2059 | 10 | <0.01 | <0.01 | <0.01 | <0.01 | >0.1 | >0.1 |
| SSP245 | 2020-2059 | 100 | <0.01 | <0.01 | <0.01 | <0.01 | >0.1 | >0.1 |
| SSP585 | 2020-2059 | 10 | <0.01 | <0.01 | <0.01 | <0.01 | >0.1 | >0.1 |
| SSP585 | 2020-2059 | 100 | <0.01 | <0.01 | <0.05 | <0.01 | >0.1 | >0.1 |
| SSP245 | 2060-2099 | 10 | <0.01 | <0.01 | <0.01 | >0.1 | >0.1 | >0.1 |
| SSP245 | 2060-2099 | 100 | <0.01 | <0.01 | <0.01 | <0.01 | >0.1 | <0.1 |
| SSP585 | 2060-2099 | 10 | <0.01 | <0.01 | <0.01 | >0.1 | >0.1 | >0.1 |
| SSP585 | 2060-2099 | 100 | <0.01 | <0.01 | <0.01 | <0.01 | >0.1 | >0.1 |


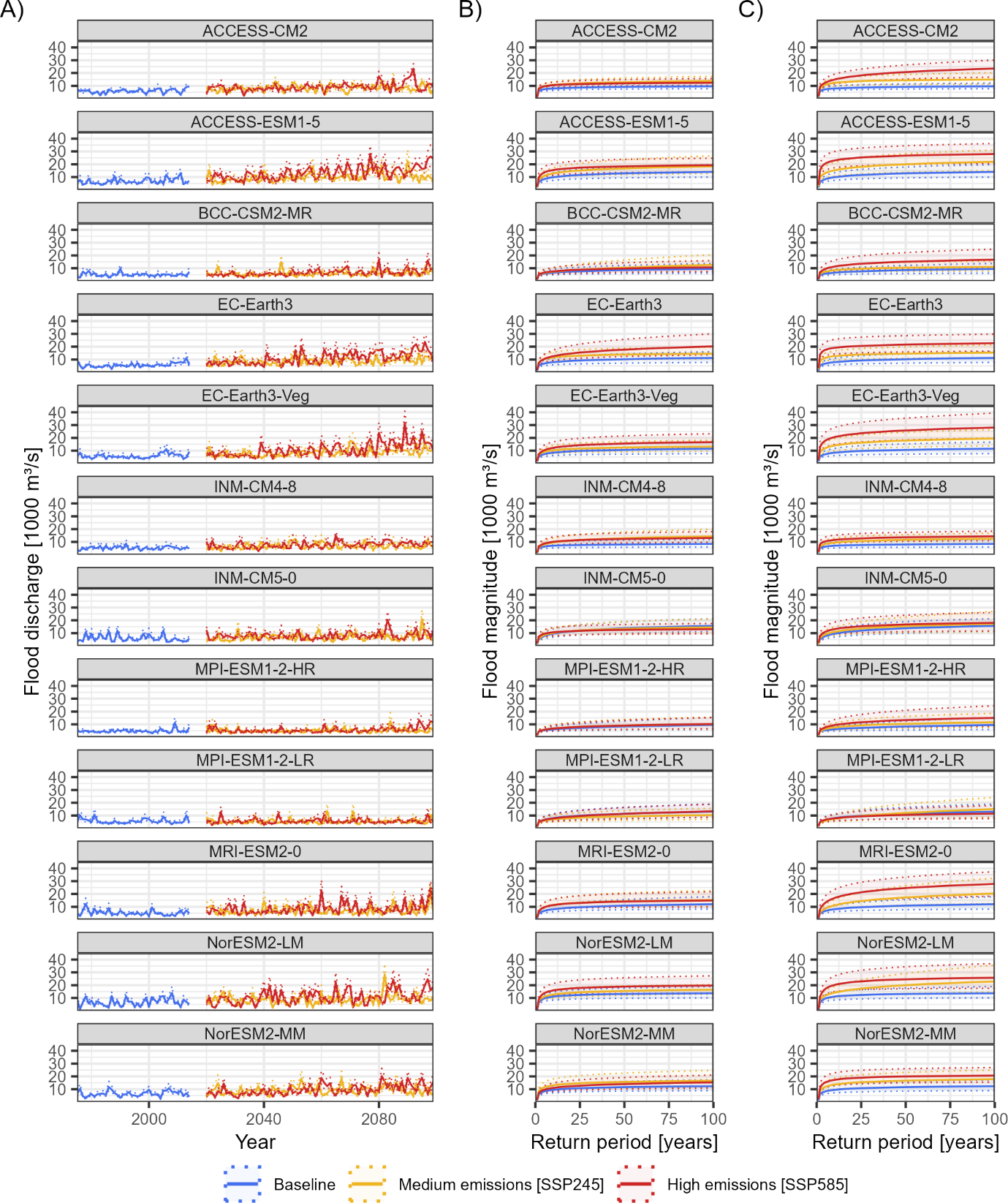


Figure S 6: The flood predictions of the 12 CMIP6 members^12^. Plot A shows the time series of the AMAX events, Plot B shows the Flood Frequency Analysis of the near-future (2020 – 2059) and Plot C shows the Flood Frequency Analysis of the far-future (2060 – 2099). The solid lines are the median predictions, and the dotted lines are the 10^th^ and 90^th^ percentiles of the ensemble.

**References**

1. Lehner, B., Verdin, K. & Jarvis, A. New global hydrography derived from spaceborne elevation data. *Eos, Transactions American Geophysical Union* **89**, 93–94 (2008).

2. Terink, W., Lutz, A. F. & Immerzeel, Walter W. *SPHY v2.0: Spatial Processes in Hydrology - Model Theory, Installation, and Data Preparation*. (2015).

3. De Boer, F. *HiHydroSoil: A High Resolution Soil Map of Hydraulic Properties. Version 1.2*. (2016).

4. Uddin, K. *et al.* Development of 2010 national land cover database for the Nepal. *Journal of environmental management* **148**, 82–90 (2015).

5. Arino, O. *et al.* Global Land Cover Map for 2009 (GlobCover 2009). *© European Space Agency (ESA) & Université catholique de Louvain (UCL)* PANGAEA https://doi.org/10.1594/PANGAEA.787668 (2012).

6. RGI Consortium, . Randolph Glacier Inventory - A Dataset of Global Glacier Outlines, Version 6. (2017) doi:10.7265/4m1f-gd79.

7. Scherler, D., Wulf, H. & Gorelick, N. Global assessment of supraglacial debris‐cover extents. *Geophysical Research Letters* **45**, 11–798 (2018).

8. Farinotti, D. *et al.* A consensus estimate for the ice thickness distribution of all glaciers on Earth. *Nature Geoscience* **12**, 168–173 (2019).

9. Weedon, G. *et al.* Creation of the WATCH forcing data and its use to assess global and regional reference crop evaporation over land during the twentieth century. *Journal of Hydrometeorology* **12**, 823–848 (2011).

10. Weedon, G. P. *et al.* The WFDEI meteorological forcing data set: WATCH Forcing Data methodology applied to ERA‐Interim reanalysis data. *Water Resources Research* **50**, 7505–7514 (2014).

11. Huffman, G., J. *et al.* *NASA Global Precipitation Measurement (GPM) - Integrated Multi-satellitE Retrievals for GPM (IMERG)*. 38 (2019).

12. Mishra, V., Bhatia, U. & Tiwari, A. D. Bias-corrected climate projections for South Asia from Coupled Model Intercomparison Project-6. *Scientific Data* **7**, 338 (2020).

13. Running, S. W., Mu, Q., Zhao, M. & Moreno, A. MODIS global terrestrial evapotranspiration (ET) product (MOD16A2/A3 and year-end gap-filled MOD16A2GF/A3GF) NASA Earth Observing System MODIS Land Algorithm (for collection 6). *National Aeronautics and Space Administration, Washington, DC, USA [data set], https://doi. org/10.5067/MODIS/MOD16A2* **6**, (2019).

14. Muhammad, S. & Thapa, A. An improved Terra–Aqua MODIS snow cover and Randolph Glacier Inventory 6.0 combined product (MOYDGL06*) for high-mountain Asia between 2002 and 2018. *Earth System Science Data* **12**, 345–356 (2020).

15. Jarvis, A., Reuter, H., Nelson, A. & Guevara, E. Hole-filled SRTM for the globe Version 4. (2008).
